# Characterization of interactions of dietary cholesterol with the murine and human gut microbiome

**DOI:** 10.1038/s41564-022-01195-9

**Published:** 2022-08-18

**Authors:** Henry H. Le, Min-Ting Lee, Kevin R. Besler, Janine M. C. Comrie, Elizabeth L. Johnson

**Affiliations:** Division of Nutritional Sciences, Cornell Univesity, Ithaca, NY USA

**Keywords:** Microbiome, Sterols, Metabolomics

## Abstract

Consumption of dietary lipids, such as cholesterol, modulates the gut microbiome with consequences for host health through the production of microbiome-derived metabolites. Despite the implications for host metabolism, a limited number of specific interactions of the gut microbiome with diet-derived lipids have been characterized. This is partially because obtaining species-level resolution of the responsible taxa can be challenging and additional approaches are needed to identify health-relevant metabolites produced from cholesterol–microbiome interactions. Here we performed bio-orthogonal labelling sort sequence spectrometry, a click chemistry based workflow, to profile cholesterol-specific host–microbe interactions. Mice were exposed to an alkyne-functionalized variant of cholesterol and 16S ribosomal RNA gene amplicon sequencing of faecal samples identified diet-derived cholesterol-interacting microbes from the genera *Bacteroides*, *Bifidobacterium*, *Enterococcus* and *Parabacteroides*. Shotgun metagenomic analysis provided species-level resolution of diet-derived cholesterol-interacting microbes with enrichment of bile acid-like and sulfotransferase-like activities. Using untargeted metabolomics, we identify that cholesterol is converted to cholesterol sulfate in a *Bacteroides*-specific manner via the enzyme BT_0416. Mice monocolonized with *Bacteroides thetaiotaomicron* lacking Bt_0416 showed altered host cholesterol and cholesterol sulfate compared with wild-type mice, identifying a previously uncharacterized microbiome-transformation of cholesterol and a mechanism for microbiome-dependent contributions to host phenotype. Moreover, identification of a cholesterol-responsive sulfotransferase in *Bacteroides* suggests diet-dependent mechanisms for altering microbiome-specific cholesterol metabolism. Overall, our work identifies numerous cholesterol-interacting microbes with implications for more precise microbiome-conscious regulation of host cholesterol homeostasis.

## Abstract (web summary)

Members of the murine and human gut microbiome can interact with dietary cholesterol. In particular, *Bacteroides* sulfotransferase activity can convert cholesterol to cholesterol sulfate and impact cholesterol levels in vivo.

## Main

Diet is a defining factor determining the function of the gut microbiome with important consequences for host well-being^1–3^. Dietary fat intake can dictate microbiome community structure^4–6^ but how different classes of fats and lipids interact with specific members of the gut microbiome to elicit changes in host health is not well understood. A better understanding of how distinct lipids interact with the microbiome will allow for a more effective use of diet in attempts to precisely modulate the function of the gut microbiome. One dietary lipid whose microbial processing has great consequences for host metabolic health is cholesterol, an animal lipid essential to a variety of biological processes, including membrane dynamics, lipid trafficking and molecular signalling^7,8^. Cholesterol is acquired from either the diet or through de novo biosynthesis by the liver^8^. Digestive systems secrete cholesterol, along with other bile constituents, into the small intestines during digestion^9^. Imbalances in cholesterol homeostasis, such as hypercholesterolaemia, have been linked to the development of atherosclerotic lesions and cardiovascular disease^10,11^.

Contributing determinants of circulating cholesterol, especially low-density lipoprotein cholesterol, include genetic and dietary factors^12–14^. The gut microbiome has also been shown to have roles in host-associated cholesterol biology, including those that modulate low-density lipoprotein cholesterol^15,16^. For example, previous work has established that the human gut microbiome can also metabolize cholesterol, where Kenny et al. characterize the conversion of cholesterol to coprostanol by gut microbes^17^. Increasing abundances of coprostanol in the stool of human subjects was linked to healthy lipid profiles, suggesting that the microbiome, via cholesterol, plays a role in regulating host processes. Comprehensive analysis identified bacterial genes responsible for coprostanol biosynthesis; however, the identity of additional bacterial genes and gut bacterial species responsible for interacting with diet-derived cholesterol remains elusive. Furthermore, although a handful of gut microbe-dependent bile acid transformations have been described to date, to our knowledge, the cholesterol–coprostanol transition appears to be the only cholesterol transformation identified for gut microbes^18–20^.

To further characterize how cholesterol interacts with the gut microbiome, we employed our recently developed method known as bio-orthogonal labelling sort sequence spectrometry (BOSSS)^21^. In this method, alkyne-labelled variants of dietary constituents are fed to mice. Bacterial caecal constituents are harvested and undergo bio-orthogonal chemical ligation to an azide-bearing fluorophore, Alexa Fluor 647-azide^22^. Thus, cholesterol-interacting microbes can be detected by their red fluorescence. Cholesterol-interacting microbes are then isolated from microbes that do not interact with cholesterol via fluorescence-activated cell sorting (FACS)^23^. Once positive interactors are isolated, sequencing analysis ascertains the identities of the associated gut microbes. Finally, comparative metabolomics of isolated microbiome samples before fluorescent labelling allows for the identification of metabolites transformed by the microbiome from exogenous cholesterol. In this work, we identify *Bacteroides* spp. as cholesterol-metabolizing microbes. Further analysis identified BT_0416 as the operative enzyme in *Bacteroides thetaiotaomicron* responsible for converting cholesterol to cholesterol-3-sulfate (Chol^Sulf^). Mice monocolonized with *Bt_0416*-knockout *B. thetaiotaomicron* showed significantly lower Chol^Sulf^ in both caecal and stool metabolomes. Blood cholesterol levels were significantly altered between mice colonized with *B. thetaiotaomicron* strains competent versus null in sulfotransferase activity. In addition, labelled microbially derived Chol^Sulf^ was detected in hepatic portal vein blood samples of conventionally raised mice, further supporting the potential for microbial Chol^Sulf^ production to directly influence host biology. Overall, our work sheds light on *Bacteroides* as an active participant in host Chol^Sulf^ metabolism and identifies a catalogue of other cholesterol-dependent gut microbiome associations that are informative for investigations of diet–microbiome interactions in multiple systems.

## Results

### Isolation of microbes that interact with diet-derived cholesterol

**Fig. 1:**
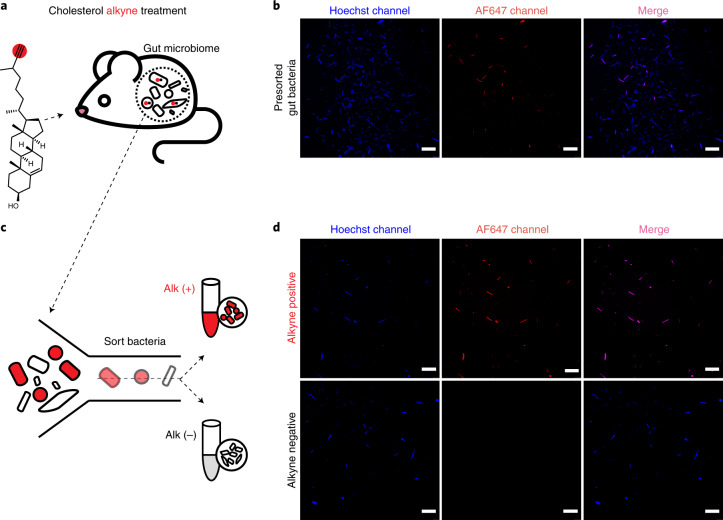
Isolation of diet-derived cholesterol-interacting microbes using bio-orthogonal labelling and FACS. **a**, Schematic showing treatment of mice with Chol^Alk^ to identify microbes that take up dietary cholesterol and derivatives of dietary cholesterol. **b**, Confocal microscopy detecting Chol^Alk^-interacting bacteria from mouse caecal contents (*n* = 6, representative images from one mouse are shown). **c**, Schematic depicting FACS-based separation of microbial communities into cholesterol-interacting (Alk+) or not interacting (Alk−) populations. **d**, Confocal microscopy detecting the presence (alkyne positive, Alk+) or absence (alkyne negative, Alk−) of Chol^Alk^ derivatives in microbiome samples separated using FACS (*n* = 4, representative images from one mouse are shown). For confocal microscopy staining: blue, Hoechst 33342; red, Alexa Fluor 647-azide (AF647). Scale bar, 20 μm.

**Extended Data Fig. 1:**
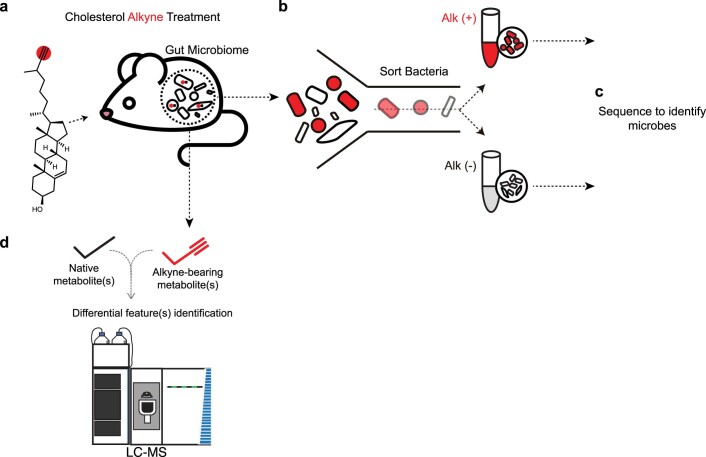
Schematic detailing the BOSSS methodology used to identify dietary cholesterol-interacting microbes and their metabolic transformations of cholesterol. **a**, An alkyne-modified form of cholesterol is orally introduced to mice which allows for the tracking of dietary cholesterol and derivatives of dietary cholesterol into the microbiome of mice. Isolated microbiome samples from caecal contents are **b**, sorted to separate interacting (Alk+) versus non-interacting (Alk-) microbes in order to **c**, identify these microbes using metagenomic sequencing. **d**, Separately, metabolomes from unsorted microbiome samples from cholesterol-alkyne exposed mice can be compared to metabolomes from mice exposed to native cholesterol to determine differential features found in the cholesterol alkyne exposed state. These features are then queried in the native cholesterol state to make sure that the transformation is not an artifact of the alkyne modification of cholesterol.

**Extended Data Fig. 2:**
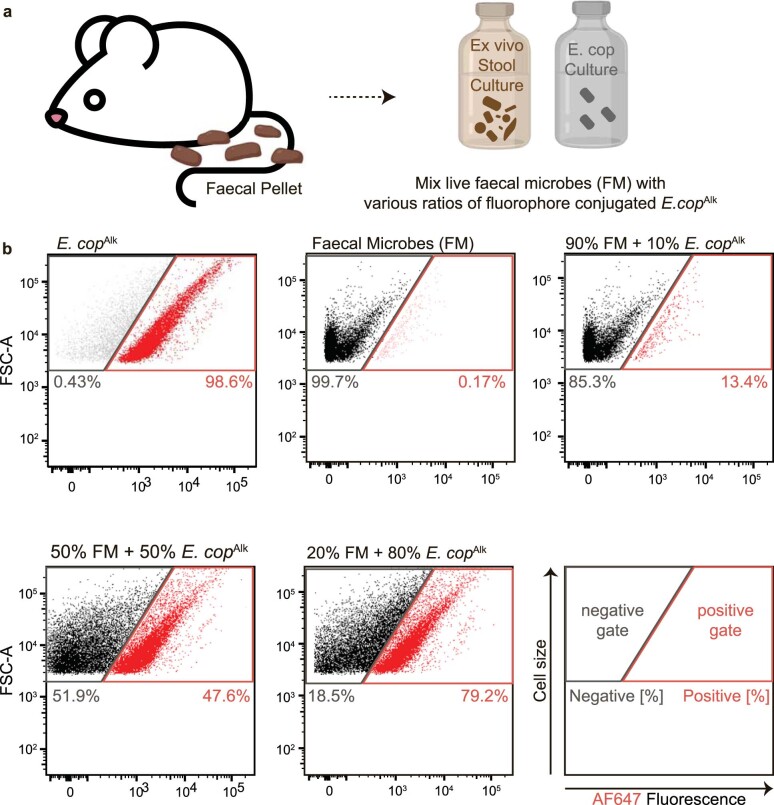
Development of FACS gates for detecting and isolating cholesterol-interacting gut microbiota using *Eubacterium coprostanoligenes*. **a**, Fecal microbes were freshly extracted from murine faecal pellets, spiked with *E. coprostanoligenes* cultured with Chol^Alk^ and click-stained with AF647-azide (*E. cop*^Alk^). **b**, Illustration of the FACS gating strategy aiming to isolate cholesterol-interacting gut microbes from complex gut microbiome populations. Representative scatter plots of different ratios of spiked-in *E. cop*^Alk^ to faecal microbes validate the gating stringency suitable for downstream FACS application on the in vivo microbial samples.

To gain a better understanding of how dietary cholesterol interacts with the gut microbiome, we exposed mice to a modified form of cholesterol (cholesterol alkyne (Chol^Alk^)) that has been readily used to investigate cholesterol metabolism in established systems^24,25^ and can be traced through diet–microbiome interactions using the BOSSS workflow^21,24^ (Extended Data Fig. 1). Chol^Alk^ was introduced orally into mice by daily gavage for a week and microbial populations were isolated from caecal contents. Oral administration of native cholesterol was also conduced as a control for any off-target effects caused by the alkyne modification that would be observed in downstream metabolomic analyses. Microbiome samples were then categorized into two populations: a population that interacts with Chol^Alk^ or any alkyne persistent metabolic derivative (Alk+); and a second population null for Chol^Alk^ interaction (Alk−). Thus, in the in vivo condition, our Alk+ fraction is inclusive of microbes that interact directly with dietary cholesterol and host (or microbial) modifications of dietary cholesterol, all of which we refer to as diet-derived cholesterol interactors. To identify these populations, we labelled Alk+ microbes with azide-bearing AF647 through the well-established copper-catalysed ‘click’ cyclo-addition reaction^22^ (Fig. 1a). Fluorescence microscopy identified that a subset of microbial caecal content were Alk+ (Fig. 1b). To further investigate the identity and metabolic capacity of dietary cholesterol-interacting microbes, we sorted diet-derived cholesterol-interacting Alk+ populations from non-interacting Alk− populations using FACS (Fig. 1c and Supplementary Table 3). To develop Alk+ and Alk− flow cytometer sorting parameters, we cultured *Eubacterium coprostanoligenes* (*E. cop*)^26^, a bacterium known to interact with cholesterol via conversion of cholesterol to coprostanol, with Chol^Alk^ (Extended Data Fig. 2). Microscopy confirmed robust Chol^Alk^ assimilation by *E. cop*. Thus, Alk+ gates were defined based on the fluorescence signal detected in *E. cop* + Chol^Alk^ samples. Alk− gates were defined using microbes isolated from unlabelled stool samples. Using parameters for Alk+ and Alk− populations, diet-derived cholesterol-interacting and non-interacting populations were isolated through FACS and it was confirmed via microscopy that populations were properly segregated (Fig. 1d). Representative FACS density plots are shown in Supplementary Fig. 1. The established Alk+ and Alk− gates were utilized for all the samples in this study and reliably identified a pure population of Alk+ dietary cholesterol-interacting bacteria (Extended Data Fig. 2).

### Identification of microbes that interact with diet-derived cholesterol

**Fig. 2:**
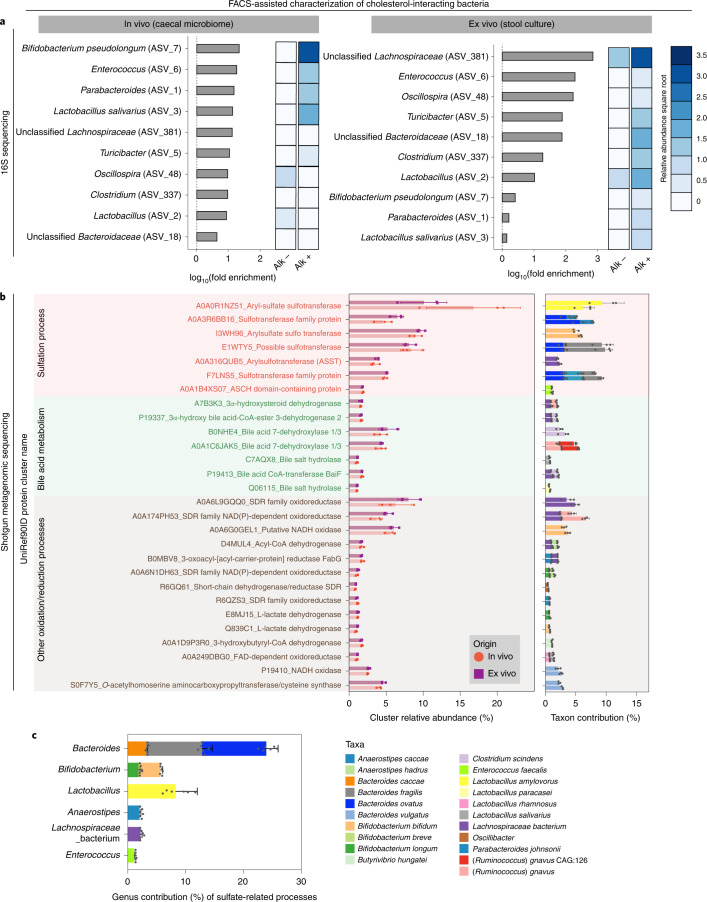
Gut microbes that interact with diet-derived cholesterol. **a**, 16S rRNA gene amplicon sequencing identifies taxonomic classification of cholesterol-interacting microbes enriched by FACS using the BOSSS workflow. The fold-enrichment is between relative abundances of taxa in the Alk+ fraction compared with the Alk− fraction and the heatmaps represent square root transformed relative abundances of the ASVs. Relative abundances of Alk+ and Alk− samples were averaged separately for both in vivo and ex vivo experiments (*n* = 3 for each sorted population). **b**, Metagenomic analysis identifies UniRef protein clusters associated with cholesterol-interacting microbes. The left bar plot demonstrates the relative abundance of each enriched cluster, in which the background is colour differentiated based on their grouped biochemical annotations, and the bar and dot colours indicate the sample source (*n* = 3 from biologically independent animals for in vivo and *n* = 3 for ex vivo biologically independent stool cultures). c.p.m., copies per million. The right bar plot shows the mean contribution of the species identified in the Alk+ to the corresponding UniRef protein clusters. Per-species cluster abundance values were normalized to each cluster within each sample individually, and the mean abundances were further taken across individuals. **c**, Per-genus contribution of all annotated sulfation processes in the Alk+, cholesterol-interacting microbiome population in both in vivo and ex vivo samples as described in **b**. Bar chart values are mean ± s.d.

Next, we sought to identify the cholesterol-interacting microbes isolated from Alk+ FACS-sorted microbiome samples. Both FACS-sorted populations (Alk+ and Alk−) (Fig. 1) and presorted input samples were first subjected to 16S ribosomal RNA gene amplicon sequencing (16S sequencing) analysis to determine the composition of the bacterial community. The Divisive Amplicon Denoising Algorithm 2 pipeline was applied to the sequencing data to improve the sensitivity of differentiating sequence variation in the identified microbial populations^27^. To avoid loss of minor cholesterol-interacting microbes, fold-enrichment of the Alk+ fraction was calculated based on the Alk− and presort inputs for each sample (Fig. 2a). From this analysis, there were ten amplicon sequence variants (ASVs) with varied taxonomic resolutions enriched in the Alk+ fraction (Fig. 2a).

**Extended Data Fig. 3:**
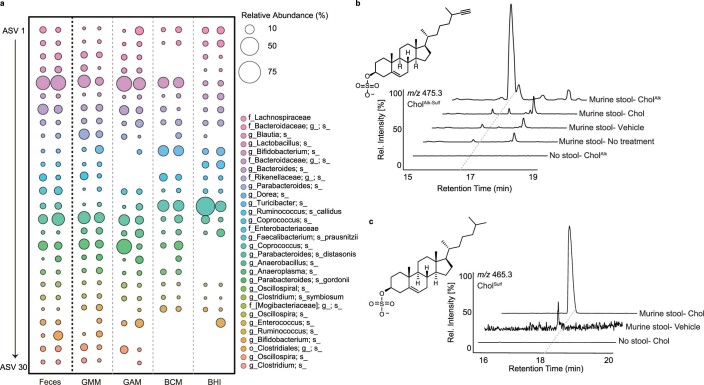
*Ex vivo* culture medium selection. **a**, The top 30 amplicon sequence variants (ASVs) of the microbial composition of the original mice faecal samples and that of 2-day *ex vivo* cultures using different media for the corresponding faecal pellets. Different colored circles represent the relative abundance of the individual ASV that are represented above 1% in the original mice faeces. **b**, Ion chromatograms of cultures containing murine stool treated with either cholesterol alkyne, cholesterol, vehicle, or no treatment, demonstrating the formation of cholesterol alkyne sulfate. **c**, Ion chromatograms of cultures containing murine stool treated with either cholesterol (chol), vehicle, or no treatment, demonstrating the formation of cholesterol sulfate.

To better understand whether dietary cholesterol interactions were dependent on host-transformations of cholesterol versus direct microbial interaction with cholesterol, we developed an ex vivo system to separate cholesterol interactions that were purely microbial from those that depended on host contributions. To initiate this process, we utilized the BOSSS pipeline on faecal cultures (Supplementary Fig. 2). Because ex vivo cultures never perfectly mimic the host gut, we utilized two different culturing conditions. Our first medium (basal cholesterol medium (BCM)) was adopted from Kenny et al., and employs conditions known to support the growth of cholesterol-utilizing *E. coprostanoligenes*^17^. A second medium (gut-mimic medium (GMM)), developed by Li et al., attempts to preserve microbiome composition and may support a wider array of cholesterol-interacting bacteria^28^. Analogous to the contents of our caecal samples, a subset of the Chol^Alk^-treated bacteria labelled positive from either medium, suggesting that some portion of the cholesterol-interacting bacteria survived ex vivo culturing (Extended Data Fig. 3). Ex vivo cultures were FACS-sorted under the same conditions as stool samples from in vivo samples for identification of cholesterol-interacting (Alk+) and non-interacting (Alk−) microbes.

We then pooled the ex vivo and in vivo 16S sequencing data based on their sort-fraction and performed differential analysis to identify interactors. The seven ASVs significantly enriched in the Alk+ fraction correspond to the taxa identified in the fold-enrichment analysis, further confirming cholesterol interactors. In general, the community profiles from the Alk+ fraction contain microbes congruent with previous investigations that analysed shifts in microbiome community composition associated with dietary cholesterol content^15,18,29–33^. *Bifidobacterium pseudolongum* was identified as the most prominently enriched bacterium in our Alk+ samples, indicating notable interaction with diet-derived cholesterol^34–36^. Microbes from the genera *Clostridium*, *Parabacteroides, Oscillospira* and *Turicibacter*, which are known to engage bile acids, were also enriched in the Alk+ populations^18,37–39^. *Enterococcus* was notably enriched in both in vivo and ex vivo systems. Studies have demonstrated that various isolates of *Enterococcus* act as carriers of cholesterol, both in vivo and in vitro, which aids in host export of cholesterol via defecation^40,41^. It is important to note that associations as measured via relative abundance and fold-enrichment differ. For instance, *Lactobacillus* (ASV3 and ASV2) was high in overall abundance, and therefore likely to have roles in cholesterol-associated activities, and its enrichment in Alk+ was not pronounced, suggesting a metabolic or spatially limited cholesterol-interacting capacity (Fig. 2a and Supplementary Fig. 3). Taken together, our FACS-guided 16S sequencing efforts identified a diet-derived cholesterol-interacting subset of the murine gut microbiome, all of which may contribute to host-relevant roles in cholesterol biology.

### Certain cholesterol-interacting microbes possess sulfotransferase activity

To improve our taxonomic identifications and map metabolic activities to microbial taxa, we performed shotgun metagenomic sequencing on the FACS-sorted microbial consortia. To understand the metabolic response of microbes that take up exogenous cholesterol, we assessed the differential gene clusters between Alk+ and Alk− (with significance threshold false discovery rate (FDR) *P* < 0.05). Differences between these two populations identified cholesterol-associated gene functions within the diet-derived cholesterol-interacting gut microbiome community. We identified 28 UniRef clusters whose abundances were significantly enriched (FDR *P* < 0.05) in the Alk+ fraction compared with the Alk− fraction (Fig. 2b, Supplementary Fig. 4 and Supplementary Table 4). The biochemical processes of the identified clusters were grouped for putative activities in the Alk+ population. Further analysis identified a subset of bacteria that harnessed sulfate-related processes, some of which tentatively have a role in the biosynthesis of Chol^Sulf^ (Fig. 2b). To this end, we then mapped the average genus-level microbial contributions to the identified sulfation-related clusters in the Alk+ sort (Fig. 2c and Supplementary Table 4). We noted that these contributors mainly reside among *Bacteroides* spp. (~25%), *Bifidobacterium* spp. (~7%), *Lactobacillus* spp. (~8%) and *Anaerostipes* spp. (~1%) (Fig. 2c).

### Untargeted metabolomics identifies a microbiome-associated cholesterol transformation

**Fig. 3:**
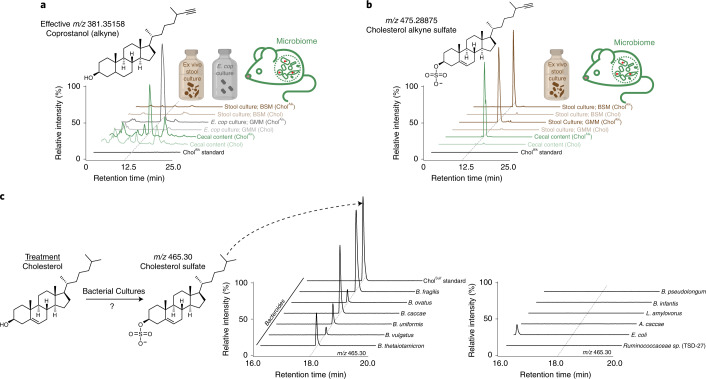
The murine gut microbiota can transform cholesterol to Chol^Sulf^. **a**, Representative structure of coprostanol alkyne. Ion chromatograms depicting detection of coprostanol alkyne from caecal content, *E. cop* cultures, and ex vivo stool cultures. **b**, Representative structure of Chol^Alk-Sulf^. Ion chromatograms depicting detection of Chol^Alk-Sulf^ in caecal content and ex vivo stool cultures. **c**, Bacterial cholesterol sulfotransferase candidates identified from metagenomic analysis were screened for Chol^Sulf^ production. Species-specific cultures were treated with cholesterol. Ion chromatograms represent the detection of Chol^Sulf^ (*n* = 2 biological replicates per species, representative traces shown here).

**Extended Data Fig. 4:**
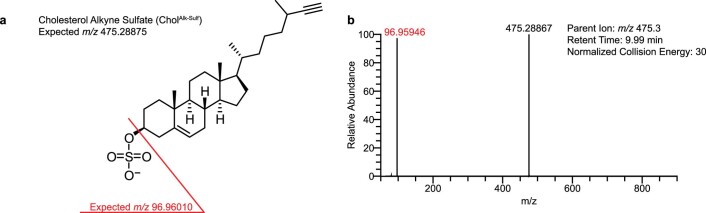
High-resolution tandem mass spectra of cholesterol alkyne sulfate illustrating sulfate fragment. **a**, MS2 fragmentation pattern of cholesterol alkyne sulfate and **b**, MS2 spectrum of cholesterol alkyne sulfate from caecal content of mice treated with cholesterol alkyne.

In tandem with FACS-assisted processing, the caecal contents of mice fed either cholesterol or Chol^Alk^ were collected and prepared as in Lee et al. for analysis via reverse-phase liquid chromatography coupled to high-resolution mass spectrometry (LC–MS)^21^. Comparative analysis was carried out utilizing the XCMS package within the Metaboseek software suite^42–44^. Several of the dominant features detected in positive mode reflected features detected in isolated *E. cop* cultures, verifying that the gut microbiome actively converts Chol^Alk^ to coprostanol^Alk^ (Fig. 3a and Supplementary Table 5). Furthermore, this establishes that Chol^Alk^ is a suitable surrogate for cholesterol to monitor transformations of the A ring, and perhaps more generally the steroid core, among gut microbes. Because in-depth validation is needed to determine true nutrient interactors identified using the BOSSS methodology, we focused on defining a previously uncharacterized microbiome-transformation of dietary cholesterol based on the following criteria: (1) the metabolic process was enriched in the gene set of diet-derived cholesterol-interacting microbes in both the in vivo and ex vivo culturing conditions; (2) the metabolic product of the transformation was detected in the in vivo and ex vivo comparative metabolomics analysis in the alkyne form (Supplementary Table 6) and also in the native cholesterol condition; and (3) genes belonging to the biosynthetic pathway could be genetically manipulated in the identified interacting species. Assessment of the caecal content analysed in negative mode revealed an upregulated molecular feature of *m/z* 475.2879 corresponding to the chemical formula C_28_H_44_O_4_S (Fig. 3b). Tandem mass spectrometry (MS/MS) analysis of the unidentified feature revealed a fragment of 96.9595, suggesting loss of sulfate (Extended Data Fig. 4). Collectively, comparative metabolomics analysis tentatively identifies the feature as alkyne-bearing Chol^Sulf^ (Chol^Alk-Sulf^), suggesting that gut microbes facilitate the conversion of cholesterol to Chol^Sulf^. We chose to focus on this microbial conversion of cholesterol to Chol^Sulf^ because it satisfies the above-mentioned criteria for a microbial transformation of dietary cholesterol that is suited for further study.

### Ex vivo stool cultures convert cholesterol to Chol^Sulf^

Because the conversion of cholesterol to Chol^Sulf^ has been previously identified as a host-transformation, we decided to perform ex vivo cultures of stool from mice to determine whether faecal microbes could also perform this transformation^45^. To initiate this process, we utilized the BOSSS pipeline on faecal cultures. Analysis of the ex vivo cultures from either culture medium showed conversion of Chol^Alk^ to Chol^Alk-Sulf^ (Fig. 3b and Extended Data Fig. 3b). Furthermore, ex vivo cultures showed efficient conversion of unlabelled cholesterol to Chol^Sulf^ (Extended Data Fig. 3c), verifying that the observed transformations were not an anomaly of the alkyne functionalization. Chol^Sulf^ from faecal cultures matched peak shape, retention time and MS/MS fragmentation patterns of Chol^Sulf^ standards. Interestingly, although we detected formation of coprostanol in caecal samples, our ex vivo cultures failed to produce coprostanol (Fig. 3a). Although many parameters may be optimized to culture coprostanol biosynthetic gut microbes, from this point forward we concentrate our attention on the capacity of the gut microbiome to make Chol^Sulf^. Sulfation of diet-, xenobiotic- and microbiome-derived metabolites had long been assumed to be a host process driven by hepatic detoxification^46^. To our knowledge, these results demonstrate a previously uncharacterized example of an exogenous metabolite sulfated by the gut microbiome.

### *Bacteroides* converts cholesterol to Chol^Sulf^

**Extended Data Fig. 5:**
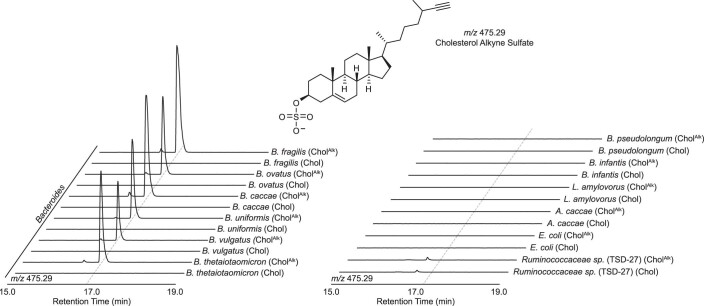
*Bacteroides* genus converts cholesterol to cholesterol sulfate. Ion chromatograms of cholesterol alkyne sulfate in cultures incubated with cholesterol (Chol) or cholesterol alkyne (Chol^Alk^) in order to determine cholesterol-interacting taxa that are able to convert cholesterol to cholesterol sulfate. *Bacteroides* species were displayed on the left to emphasize their ability to convert cholesterol to cholesterol sulfate, with other bacterial strains that failed to do the conversion arranged on the right.

**Extended Data Fig. 6:**
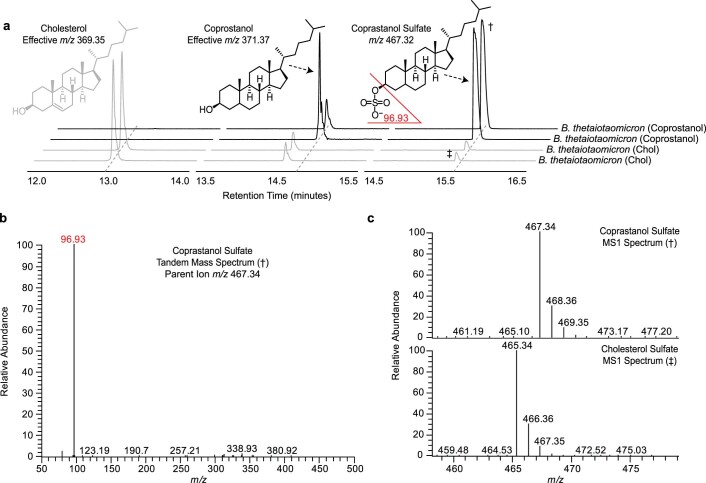
Coprostanol can be sulfated by *Bacteroides thetaiotaomicron*. **a**, Ion chromatograms and **b**, tandem mass spectra of axenic cultures of *B. thetaiotaomicron* with either cholesterol or coprostanol revealing that coprostanol can also be sulfated by *B. thetaiotaomicron* to produce coprostanol sulfate. **c**, MS1 spectra of coprstanol sulfate (†) and cholesterol sulfate (‡) demonstrating the parent ion m/z difference between the two metabolites. (†) and (‡) denote coprostanol sulfate and cholesterol sulfate respectively.

Metagenomic analysis identified a handful of candidates for Chol^Sulf^ biosynthesis. To screen for Chol^Sulf^ biosynthetic candidates, we cultured *Bacteroides ovatus, Bacteroides fragilis*, *Bifidobacterium longum* subsp. *infantis*, *Bifidobacterium pseudolongum,* *Lactobacillus* *amylovorus*, *Anaerostipes* *caccae* and *Ruminococcaceae* sp. with either cholesterol or Chol^Alk^. Targeted metabolomic analysis revealed that *Bacteroides* could convert cholesterol or Chol^Alk^ to Chol^Sulf^ or Chol^Alk-Sulf^, respectively, whereas non-*Bacteroides* candidates failed to convert Chol to Chol^Sulf^ (Fig. 3c and Extended Data Fig. 5). To determine whether these events were a generalized *Bacteroides* process, we then screened *B. caccae*, *B. uniformis*, *B. vulgatus* and *B. thetaiotaomicron*. Indeed, all *Bacteroides* successfully converted cholesterol to Chol^Sulf^ (Fig. 3c). In addition, because bile acids are also known to be sulfated, we cultured *B. thetaiotaomicron* with glycocholic acid, glycoursodeoxycholic acid, cholic acid, deoxycholic acid and lithocholic acid^47^. Both primary and secondary bile acids (Supplementary Figs. 5 and 6) tested in our study failed to be converted to their corresponding sulfated counterparts, suggesting that cholesterol is the preferred substrate. Interestingly, coprostanol, the microbiome-derived cholesterol-derivative, was also converted to its corresponding sulfate (Extended Data Fig. 6), which suggests metabolic interplay between coprostanol producers and *Bacteroides*, because *Bacteroides* themselves do not convert cholesterol to coprostanol^17^. Finally, because cholesterol and sphingolipids are commonly found in tandem through nature, we hypothesized that sphingolipids might play a role in converting cholesterol to Chol^Sulf^^48^. In light of this, we cultured a sphingolipid-synthesis null mutant of *B. thetaiotaomicron* with cholesterol; however, end-point analysis found no significant difference in Chol^Sulf^ production, showing that sphingolipid production is not required to produce Chol^Sulf^ (Supplementary Fig. 7).

**Fig. 4:**
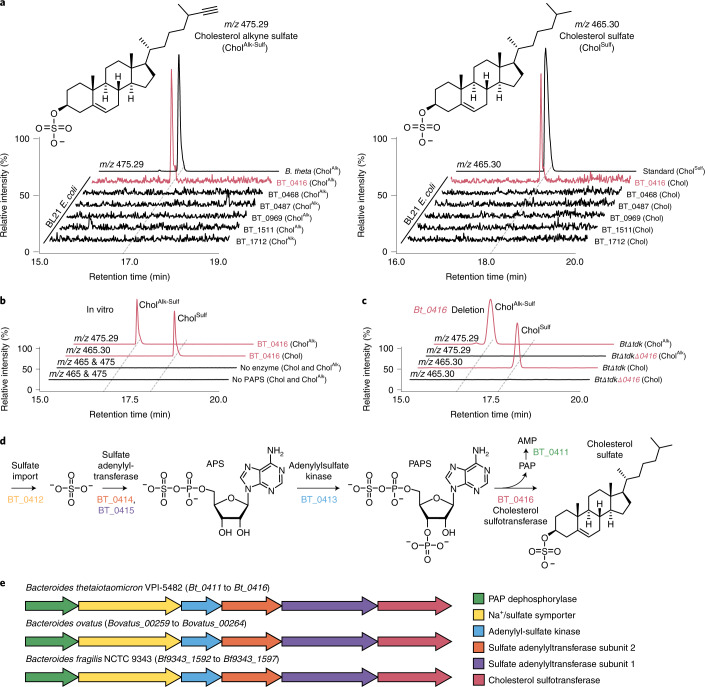
A gene cluster in *Bacteroides* converts cholesterol to Chol^Sulf^. **a**, Heterologous expression of cholesterol sulfotransferase candidates in BL21 *E. coli* treated with either Chol^Alk^ or cholesterol and ion chromatograms representing the detection of Chol^Alk-Sulf^ or Chol^Sulf^, respectively (*n* = 2 biological replicates per gene candidate, representative traces are shown here) **b**, In vitro conversion of cholesterol to Chol^Sulf^ and Chol^Alk^ to Chol^Alk-Sulf^ via enriched His_6__BT_0416 (*n* = 2 biological replicates per condition, representative traces are shown here) **c**, Ion chromatograms demonstrating that the *Bt_0416* deletion mutant does not make Chol^Sulf^. **d**, Putative biosynthesis of PAPS followed by the biosynthesis of Chol^Sulf^ in *Bacteroides*. Gene products are coloured according to the key in **e**. **e**, Biosynthetic clusters representing putative Chol^Sulf^ biosynthesis genes in *B. thetaiotaomicron*, *B. ovatus* and *B. fragilis*. Chromatograms are scaled to the largest peak in each dataset. The red text highlights metabolic functions attributed to BT_0416. Source data

**Extended Data Fig. 7:**
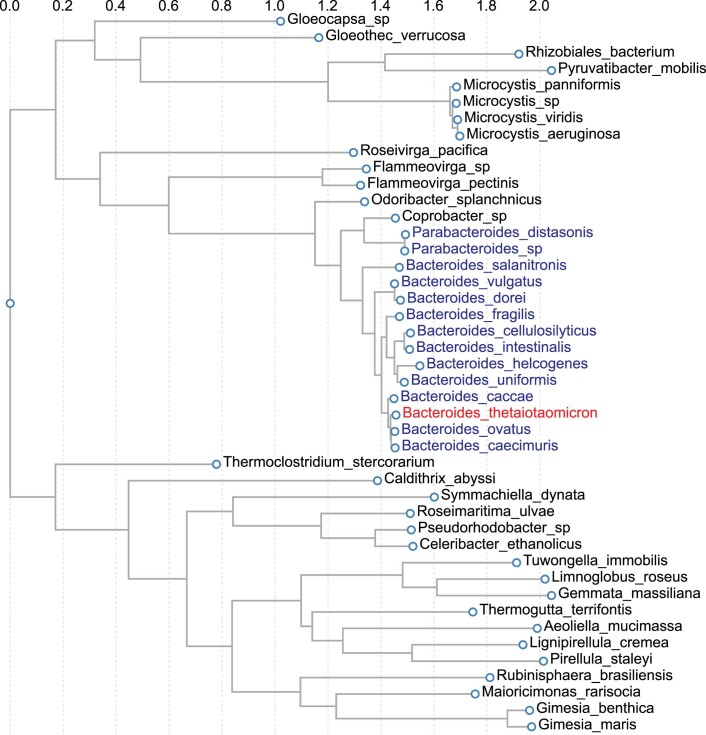
Phylogenetic tree representing organisms which contain *Bt_0416* or *Bt_0416*-like genes. Red highlights *Bt_0416* and blue highlights others *Bacteroides* species. The BLASTP analysis was performed using the KEGG BLAST tool with the top 50 resulted selected and subjected to the TREE function. Sequences were aligned using MAFFT and phylogenetic inferences were obtained using the maximum likelihood method within the tool. Scale denoted on the top of the plot indicates the nucleotide sequence divergence.

To identify the biochemical machinery within *Bacteroides* responsible for producing Chol^Sulf^, we sought out homologues of human SULT2A1 and SULT2B1, two enzymes previously identified to synthesize Chol^Sulf^^45^. BLAST analysis of *B. thetaiotaomicron*, a genome-sequenced and genetically manipulable representative *Bacteroides*^49^, identified several sulfotransferase candidates, six of which we selected for additional characterization (Fig. 4a). We focus on *B. thetaiotaomicron* as a representative to investigate *Bacteroides* cholesterol metabolism because of our validation of its ability to transform cholesterol to Chol^Sulf^ and our ability to manipulate putative Chol^Sulf^ synthesis genes in this microbe. Candidates were amplified from the *B. thetaiotaomicron* genome and cloned into the pET28 *Escherichia coli* expression vectors, maintaining the N-terminal polyhistidine tag. Cultures of BL21 *E. coli* transformed with the cholesterol sulfotransferase candidates were then treated with either cholesterol or Chol^Alk^. Because *E. coli* produces 3′-phosphoadenosine-5′-phosphosulfate (PAPS), the second putative substrate required for Chol^Sulf^ production, we envisioned live *E. coli* would complete Chol^Sulf^ biosynthesis in vivo without additional intervention^50^. Indeed, targeted analysis of our recombinantly expressed candidate cultures revealed that BT_0416 converted cholesterol to Chol^Sulf^ (Fig. 4a). Immobilized metal-affinity chromatography-enriched polyhistidine-tagged BT_0416 also reconstituted activity in vitro (Fig. 4b and Supplementary Fig. 8). Finally, deletion of *Bt_0416* from *B. thetaiotaomicron* abolished cholesterol sulfotransferase activity in vivo (Fig. 4c). Analysis of neighbouring genes identified *Bt_0411* to *Bt_0415* as putative PAPS biosynthetic enzymes. We envision that BT_0412 imports sulfate from the environment, perhaps from the catabolism of sulfated glycans, where BT_0413 to BT_0415 complete PAPS biosynthesis (Fig. 4d)^51,52^. BT_0416 then utilizes PAPS in tandem with cholesterol to generate Chol^Sulf^. Analysis of *B. ovatus* and *B. fragilis* genomes yielded identical biosynthetic clusters, suggesting conserved Chol^Sulf^ biosynthesis among *Bacteroides* (Fig. 4e and Extended Data Fig. 7).

### Microbiome sulfotransferase activity alters host Chol^Sulf^ and cholesterol levels

**Fig. 5:**
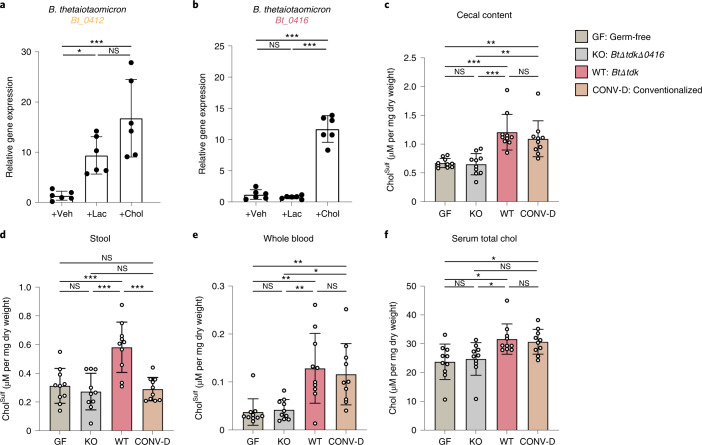
*B. thetaiotaomicron* sulfotransferase activity influences host Chol^Sulf^ and cholesterol levels in a gnotobiotic mouse model. **a**,**b**, Expression of *Bt_0412* (**a**) and *Bt_0416* (**b**) from *B. thetaiotaomicron* cultured in 10% tryptone, followed by treatment with vehicle (Veh), lactose (Lac) or cholesterol (Chol) (*n* = 6 per condition). **c**–**f**, Targeted metabolomics performed on GF mice, GF mice monocolonized with *B. thetaiotaomicron* harbouring either BT_0416-competent (WT: *BtΔtdk*) or -null (KO: *BtΔtdkΔ0416*) strains, and CONV-D mice (*n* = 10 per condition). Chol^Sulf^ measurements from caecal content (**c**), stool (**d**) and whole blood (**e**) of GF, KO, WT and CONV-D mice. **f**, Serum total cholesterol measurements of GF, KO, WT and CONV-D mice. Bar chart values are mean ± s.d. and statistical analyses were performed using one-way analysis of variance with Tukey’s multiple comparison correction. NS, *P* > 0.05; *P ≤ 0.05; **P ≤ 0.01; ***P ≤ 0.001.

**Extended Data Fig. 8:**
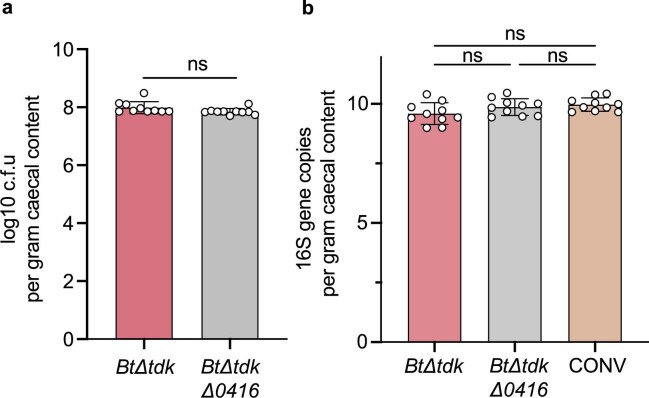
Colonization of germ-free mice orally inoculated with strains of *B. thetaiotaomicron*. **a**, Monoassociation colonization efficiencies of *Bt*Δ*tdk* and *Bt*Δ*tdk*Δ*0416* as measured via stool sample colony forming units (c.f.u) (n = 10, *P* > 0.05: ns, determined by a two-sided Student’s t-test). **b**, Bacterial load in the cecal content of monocolonized and conventionalized mice as measured by 16 S rDNA copies (n = 10, *P* > 0.05: ns, determined by one-way ANOVA with Tukey posttest). Source data

To test how microbial sulfotransferase activity contributes to host–microbiome interactions, we first attempted to understand whether the expression of *Bacteroides* sulfotransferase activity was responsive to exogenous cholesterol. Previous work by Bjursell et al. identified that the PAPS synthesis genes *Bt_0412* to *Bt_0415* were upregulated in suckling mice, suggesting that PAPS biosynthesis could be modulated^51^. To mimic their in vivo results, in vitro analysis of *B. thetaiotaomicron* was cultured in 10% tryptone with or without lactose, the former being the condition that increased expression of the PAPS cluster. Because *Bt_0416* is immediately adjacent to the putative PAPS cluster, we wished to determine whether *Bt_0416* was also induced with lactose and/or cholesterol via a quantitative polymerase chain reaction with reverse transcription (RT–qPCR). Although lactose did indeed induce *Bt_0412* (Fig. 5a), *Bt_0416* was unaltered (Fig. 4b). Interestingly, treatment with cholesterol significantly upregulated both genes, suggesting convergent regulatory modes of PAPS and Chol^Sulf^ biosynthesis via cholesterol (Fig. 5a,b). After identifying that *Bt_0412* and *Bt_0416* were cholesterol-responsive genes, we then wished to determine whether BT_0416 had a role in converting cholesterol to Chol^Sulf^ and influencing host Chol^Sulf^ levels in vivo. To initiate this process, we monocolonized germ-free (GF) mice with either sulfotransferase competent (*BtΔtdk*) or null (*BtΔtdkΔ0416*) strains of *B. thetaiotaomicron*. Colonization efficiencies were not significantly different between the two strains, as determined by stool colony-forming unit (c.f.u.) counts and 16S rDNA for caecal bacterial load (Extended Data Fig. 8). Two weeks post-colonization, mice were killed, and samples were collected. Colonization of GF mice with *BtΔtdk* (wild-type (WT)) resulted in significant elevation of Chol^Sulf^ in caecal contents (Fig. 5c) and stool samples (Fig. 5d), both of which are either comparable with or higher than the conventionalized mice (CONV-D), indicating that gut bacteria are major contributors to both gut and faecal Chol^Sulf^. To determine whether gut *Bacteroides*-dependent production of Chol^Sulf^ could influence systemic host biology, we measured Chol^Sulf^ in whole blood. Indeed, mice associated with *BtΔtdkΔ0416* (knockout (KO)) had lower levels of whole-blood Chol^Sulf^ (Fig. 5e) than those associated with *BtΔtdk* (WT), uncovering a previous uncharacterized microbiome-associated perturbation in steroid metabolism. Because steroid and steroid sulfate metabolism are often linked, we decided to determine whether total serum cholesterol was altered between BT_0416-null and -competent *Bacteroides* mono-associations^53^. In agreement with previous reports^54^, lack of a microbiome led to a significant reduction in serum cholesterol in GF mice compared with CONV-D mice (Fig. 5f). We also noted that in addition to having lower serum cholesterol than mice harbouring BT_0416 activity (*BtΔtdk*), *BtΔtdkΔ0416*-colonized mice displayed a low cholesterol level similar to that in GF mice, indicating that the conversion of cholesterol to Chol^Sulf^ via the gut microbiota impacts both Chol^Sulf^ and cholesterol homeostasis.

### *B. thetaiotaomicron* contributes Chol^Sulf^ to host tissue via hepatic portal vein circulation

**Fig. 6:**
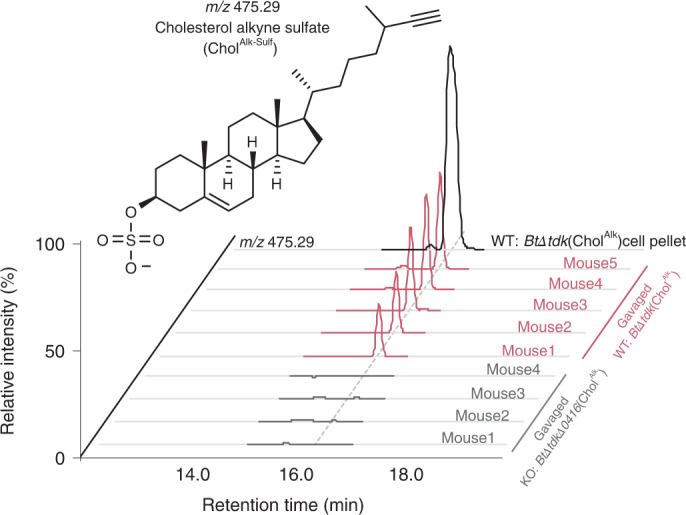
*B. thetaiotaomicron*-derived Chol^Sulf^ is taken up into hepatic portal vein blood circulation. Ion chromatograms of Chol^Alk-Sulf^ in a *B. thetaiotaomicron* strain that produces Chol^Alk-Sulf^ (WT: *BtΔtdk*(Chol^Alk^) cell pellet, black), mice exposed to a *B. thetaiotaomicron* strain null in sulfotransferase activity (KO: *BtΔtdkΔ0416*, grey; *n* = 4), and mice exposed to WT sulfotransferase competent *B. thetaiotaomicron* (WT: *BtΔtdk*, pink; *n* = 5).

To understand how microbiome production of Chol^Sulf^ contributes to host metabolism in a mouse model with conventional cholesterol metabolism, we labelled Chol^Sulf^ in *B. thetaiotaomicron* cultures using Chol^Alk^ and introduced these strains to mice orally. We have used this technique previously to understand the transfer of palmitic acid-derived bacterial lipids to host tissue^55^. The *BtΔtdk* (WT) strain readily produces Chol^Alk-Sulf^, whereas the sulfonotransferase-null strain, *BtΔtdkΔ0416* (KO), does not. When introduced orally into mice, *B. thetaiotaomicron*-derived Chol^Alk-Sulf^ was observed in the hepatic portal vein circulation of WT-gavaged mice and not in KO-gavaged mice (Fig. 6). This observation suggests that microbiome-dependent transformations of dietary cholesterol can influence host pathways through direct interaction with bacterially derived Chol^Sulf^.

## Discussion

Although Chol^Sulf^ was isolated from human samples more than 50 years ago, its origins were largely presumed to be from host processes^56^. Using BOSSS, a method for tracing dietary lipids, we determined that a limited subset of gut microbes associate with diet-derived cholesterol. Untargeted metabolomics, in combination with in vivo and ex vivo studies, revealed that cholesterol is converted to Chol^Sulf^ by microbial processes. Furthermore, culturing efforts guided by our metagenomic analysis, showed that *Bacteroides* was responsible for sulfating cholesterol, highlighting a previously uncharacterized origin for Chol^Sulf^. Interestingly, coprostanol was also sulfated by *Bacteroides* in axenic culture (Extended Data Fig. 6). Targeted analysis of caecal content metabolomes from mice treated with Chol^Alk^ identified coprostanol alkyne sulfate (Supplementary Fig. 9), suggesting cross-talk between coprostanol-forming microbes and *Bacteroides*, a metabolic network that warrants future investigation. Finally, mice mono-associated with *Bacteroides* lacking cholesterol sulfotransferase activity showed significant changes to caecal, faecal and blood Chol^Sulf^ levels, validating that the microbiome plays an important role, previously unidentified role in metabolizing exogenous cholesterol. Moreover, tracing Chol^Alk-Sulf^ uptake from *B. thetaiotaomicron* suggests that the microbiome can contribute directly to host Chol^Sulf^ levels.

**Extended Data Fig. 9:**
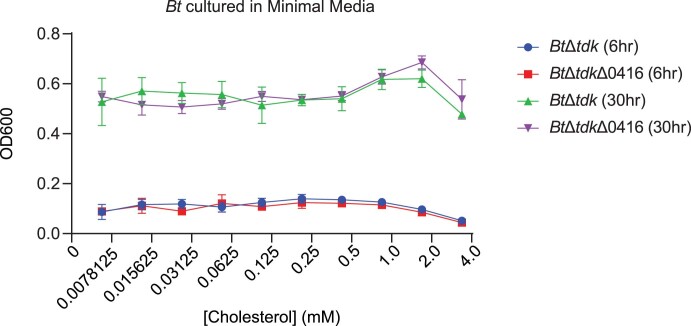
Exogenous cholesterol does not differentially affect growth of *Bt*Δ*tdk*Δ*0416* as compared to *Bt*Δ*tdk*. OD_600_ of *Bt*Δ*tdk* or *Bt*Δ*tdk*Δ*0416* cultures in minimal media supplemented with 1% Tween 80 (to increase Chol solubility) treated with various concentrations of Chol. The highest Chol concentration represents the saturation point for Chol for the culture medium. Values are the mean ± s.d. (n = 3 biologically independent cultures per condition for each concentration). Source data

The potential implications for host biology of the modulation of Chol^Sulf^ by the gut microbiome are broad. Chol^Sulf^ has been shown to inhibit 3-hydroxy-3-methylglutaryl coenzyme A reductase, effectively reducing steroid synthesis^57,58^. Chol^Sulf^ has also been shown to inhibit pancreatic elastase, an enzyme responsible for aiding the digestion of various dietary components^59^. The inhibition of pancreatic elastase via Chol^Sulf^ in the gut may suggest modulation of digestion via *Bacteroides*. In addition, Chol^Sulf^ has been shown to interact with white blood cells^60–62^ to elicit effects on the immune response^63–65^. Because *Bacteroides* do not make cholesterol, the sulfation of cholesterol appears to be an adapted role. In vitro analysis of *BtΔtdk* and *BtΔtdkΔ0416* using media saturated with cholesterol found no differences in viability, showing that the production of Chol^Sulf^ is not a scheme for cholesterol detoxification (Extended Data Fig. 9). Overall, these results suggest Chol^Sulf^ biosynthesis by *Bacteroides* may modulate the host state via numerous targets that warrant further investigation.

Limitations of the BOSSS approach include the current inability to fully differentiate between direct dietary cholesterol and diet-dependent cholesterol interactions that involve a transformation before engaging with the microbiome. In this study, this limitation is partially overcome by using ex vivo cultures to eliminate host-transformations of cholesterol as a factor. Moreover, cholesterol interactions identified by BOSSS are strengthened by validation in both microbial and host systems as was done for the Chol^Sulf^ transformation in this study. Although we chose to focus on the rich biology associated with the microbiome’s transformation of cholesterol to Chol^Sulf^, there are multiple opportunities to elucidate new biology by further exploring cholesterol-interacting microbes identified through the BOSSS pipeline. It is also of note that a native metabolite control is necessary in the BOSSS workflow to confirm that processes identified using modified metabolites are also observed in the natural state.

**Extended Data Fig. 10:**
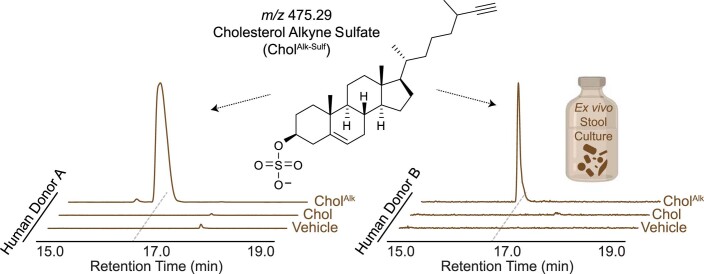
Human gut microbes convert cholesterol to cholesterol sulfate. Ion chromatograms of cholesterol alkyne sulfate demonstrating that *ex vivo* cultures of stool from human donors can convert cholesterol alkyne (Chol^Alk^) to cholesterol alkyne sulfate (Chol^Alk-Sulf^).

Our data-rich explorations included 16S rRNA gene amplicon and shotgun metagenomic sequencing, both of which indicate complex cholesterol-specific interactions. Lingering questions persist, including those involving the microbial conversion of cholesterol to coprostanol and further exploration of the functional effects of cholesterol-interacting microbes revealed through the BOSSS workflow. Finally, cultures of human stool also showed conversion of cholesterol to Chol^Sulf^ (Extended Data Fig. 10), suggesting an intriguing mechanism of cholesterol homeostasis in humans that merits further investigation. In light of this, recent studies have identified unique steroid sulfates associated with bariatric surgery that modulate glucose tolerance in a microbiome-dependent manner^66,67^. Taken together, our work demonstrates a distinct facet of cholesterol-specific microbiome biology, and the various cholesterol interactions identified in this work could reveal additional steroid-specific gut microbiome paradigms.

## Methods

### Chemicals

All HPLC solvents were purchased from Fisher Chemicals as Optima LC–MS grade. 27-Alkyne cholesterol (Chol^Alk^) was purchased from Click Chemistry Tools. Sodium cholesteryl sulfate (Chol^Sulf^), glycocholic acid, glycoursodeoxycholic acid, cholic acid, chenodeoxycholic acid and lithocholic acid were purchased from Sigma-Aldrich. Cholesterol was purchased from MP Biomedicals. Olive oil was purchased from Spectrum Chemical.

### Single-species bacterial cultures supplemented with cholesterol or Chol^Alk^

Cholesterol, Chol^Alk^ and bile acid stocks were prepared as 25 mM solutions in ethanol. Bacteria were cultured in their respective media to stationary phase. Then 1 ml of the bacterial stock was diluted in 25 ml of fresh media containing 10 μM of the respective small molecule for 48 h at 37 °C. *E. coprostanoligenes* ATCC51222 was cultured in BCM without cholesterol as described in Kenny et al.^17^. *Bacteroides* species were cultured in minimal media as described in Lee et al.^21^. *A. caccae* and *Ruminococcaceae* species (TSD-27) were cultured with ATCC medium 2971. *Bifidobacterium* species were cultured in BSM broth (Sigma-Aldrich). *Lactobacillus* species were cultured in MRS broth (Sigma-Aldrich). BL21 *E. coli* was cultured in M9 minimal media with glucose. Except for *E. coli*, all bacterial cultures were grown in glass bottles under anaerobic conditions. *E. coli* cultures were grown in sterilized Erlenmeyer flasks with shaking at 250 r.p.m. All bacterial pellets were collected at 4,000*g* for 20 min at 4 °C in 50 ml centrifuge tubes, the supernatant was aspirated, and pellets resuspended in 1 ml of PBS. The bacteria were then moved to 1.5 ml centrifuge tubes and pelleted again. The supernatant was removed and the pellet was washed once more with PBS. The spent PBS was removed, the pellet was flash-frozen using liquid nitrogen, and dried via lyophilization until further processing.

### Animal experiments

All mouse experiments were performed according to a protocol approved by the Cornell University Institutional Animal Care and Use Committee (protocol no. 2010-0065).

### In vivo dietary cholesterol uptake

Female conventionally raised excluded flora Swiss Webster mice were purchased from Taconic Biosciences at 5 weeks of age and were acclimated for at least 5 d before undergoing any procedure. For sequencing experiments, 12 mice were preweighed and randomly assigned to one of four treatment groups: (1) +cholesterol, (2) +Chol^Alk^, (3) +vehicle and (4) no gavage. Mice were housed three per cage with three mice per treatment group. For comparative metabolomics experiments, 12 mice were preweighed and randomly assigned to one of three treatment groups: (1) +cholesterol, (2) +Chol^Alk^ and (3) +vehicle. Mice were housed four per cage with between two and four mice per treatment group. Mice were housed in a climate-controlled environment with 12 h light and dark cycles and reared on a standard sterilized breeder diet (LabDiet 5021) with ad libitum access to autoclaved water. For the dietary cholesterol treatment as a part of the BOSSS workflow, mice received 100 μl of cholesterol or Chol^Alk^ at 20 mg per kg body weight in olive oil as a vehicle via oral gavage using a 20 Gauge gavage needle (Fine Science Tools). Mice were gavaged once daily with either of the two cholesterol treatments, vehicle control or no gavage for 7 d. On the day they were killed, mice received the final gavage and were then fasted for 5 h before euthanasia via decapitation. Caecal contents were collected, snap-frozen using liquid nitrogen, and stored at −80 °C until further processing. This experiment and subsequent analysis were repeated and representative metagenomic data are displayed in Fig. 2.

### Isolation and fixation of microbial cells from caecal content samples

The general procedure to isolate microbial cells from caecal content samples was modified from our workflow demonstrated in Lee et al.^21^. In brief, the thawed caecal content samples were preweighed and diluted ten times with ice-cold sterile 1× PBS in a 2-ml screw-top tube. Samples were then subjected to intermittent vortex for 5 min followed by mild sonication for 20 s total on time, with alternating 2 s on pulses, and 2 s off pulses, at 3–6 W (Qsonica Ultrasonic Processor, model Q700, with a water bath adaptor, model 431C2). Samples were centrifuged at room temperature for 2 min at 200*g*, and supernatants were transferred to a fresh 1.5 ml tube. The above steps were repeated twice on the remaining cell pellets for maximizing cell recovery and the supernatants were pooled and centrifuged at 18,000*g* for 10 min at room temperature. The bacterial pellets were washed three times with ice-cold 1% BSA/PBS. Cell pellets were resuspended in 10% buffered formalin for 10 min. Cells were then washed and incubated with 0.1% Triton X-100 in PBS for 30 min at room temperature on an end-over-end rotor for cell permeabilization and removal of non-assimilated free Chol^Alk^.

### Copper(I)-catalysed azide-alkyne cycloaddition staining (click reaction)

Bacterial cells containing Chol^Alk^ derivatives were detected and labelled with AF647-azide using freshly prepared click reaction cocktail following the manufacturer’s instructions for the Click-&-Go Click Chemistry Reaction Buffer Kit (Click Chemical Tools) at a final fluorophore concentration of 5 μM for 1 h at room temperature and in the dark. Cell pellets were washed five times and resuspended with 1% BSA/PBS to remove any non-specific fluorescent signals before fluorescence imaging and flow cytometry.

### Isolation of live gut microbes from mice faeces

Freshly collected faecal pellets from untreated mice receiving a chow diet were weighed and put into a screw-top tube filled with 250 μl of prewarmed (37 °C) and prereduced sterile PBS with 0.1% l-cysteine. For flow cytometry, the faecal microbes were isolated following the same procedure as the preparation steps for caecal microbial cells described above. For ex vivo cultures mentioned below, the samples were vortexed for 5 min and brought into an anaerobic chamber (gas mix: 70% N_2_, 25% CO_2_, 5% H_2_). The suspension was left standing for 5 min to precipitate the insoluble particles, and the supernatant portion was transferred to a fresh prereduced tube for the subsequent ex vivo culture.

### Ex vivo cultures of live faecal microbes for medium selection

A 50 μl aliquot from the isolated faecal microbial samples was used to inoculate 25 ml of four different media formulations: GMM (developed by Li et al.^28^), modified Gifu Anaerobic medium (HyServe), BCM and Brain Heart Infusion. Cultures were incubated at 37 °C in an anaerobic chamber for 2 d, and the cell pellets were harvested by centrifugation at 4,000*g* for 30 min at 4 °C.

### Ex vivo culture of live faecal microbes with cholesterol or Chol^Alk^

After identifying the top two candidate medium to culture cholesterol-interacting bacteria, 50 μl of the isolated faecal microbial samples was inoculated into 25 ml of the selected media with 10 μM cholesterol or Chol^Alk^ for 48 h under anaerobic conditions at 37 °C.

### FACS gate optimization

To optimize the capture of Chol^Alk^-interacting microbes, *E. cop*, a bacterium active in cholesterol metabolism^17^, was selected to establish the positive gate (Alk+). *E. cop* was cultured with Chol^Alk^ for 3 d (*E. cop*^Alk^), followed by AF647-azide detection as described above. The purity of the positive sort was further confirmed by both fluorescence imaging and targeted metabolomics. Unlabelled faecal microbiota were used for development of the negative gate (Alk–). To improve stringency of the gates, both *E. cop*^Alk^ and faecal microbes were first stained with 1 μg ml^−1^ Hoechst 33342 (Invitrogen) to normalize cell counts via a Leica DM500 fluorescence microscope according to Hedal et al.^68^. Before flow cytometry, the normalized cell suspensions of *E. cop*^Alk^ and faecal microbes were pooled at varying ratios and analysed using a BD Biosciences Melody Sorter. Data were acquired using BD FACSChorus RUO software v.2.0 and analysed using FlowJo v.10.6.2 software. The BD Biosciences Melody Sorter was sterilized by runs of 70% ethanol, and the instrument was kept under sterile conditions during sorting using sheath fluid prepared with sterile PBS. Samples were passed through a 35 µm nylon mesh cell strainer (Corning), the AF647-azide dye was excited using a 640-nm red laser and fluorescence was captured with a 660 nm/20 nm filter. Gates were established and adjusted following the procedures as described in Lee et al.^21^.

### FACS to identify Chol^Alk^-containing bacteria

FACS was performed as described in Lee et al.^21^ via the established gates (vida supra). Some 60,000 events in each gate with an AF647 signal greater than ~10^3^ fluorescent intensity (Alk+) or 10^2^ to 10^−2^ fluorescent intensity (Alk–) were captured and segregated into two separate 5-ml round-bottom tubes (BD Biosciences) containing 1% sterile BSA/PBS. A second round of sorting was performed on both the Alk+ and Alk– fraction with the parameters above to ensure the purity of the cells. After sorting, cells were transferred to 1.5 ml tubes and centrifuged at 18,000*g* for 10 min at 4 °C. The supernatant was discarded, and the sorted cells were counterstained with 1 μg ml^−1^ Hoechst 33342 (Invitrogen), then washed and resuspended with 1% BSA/PBS for imaging or sequencing.

### Fluorescence microscopy

Resuspended cells were mounted onto glass slides with Vectashield Vibrance mounting media (Vector Labs) and analysed using a Leica DM500 fluorescence microscope. All the images were acquired using LAS X v.3.6 software and analysed using Fiji Image J software as described in Schindelin et al.^69^. In addition, the supernatant from each microbial isolation from caecal contents was imaged for confirmation of the absence of bacteria with Hoechst 33342 (1 μg ml^−1^) and imaged using fluorescence microscopy. Samples were imaged by using two filters: UV (359 nm/461 nm) for Hoechst 33342, and Cy5 (650 nm/670 nm) for AF647 to detect the presence of alkyne-containing metabolites.

### 16S rRNA gene amplicon sequencing and analysis

Genomic DNA was isolated from the microbial cell pellets as described previously in Lee et al.^21^. The amplicon libraries were created by PCR amplification of the V4 variable region using primers with common adaptor sequences: 515F and 806R^70^. Barcoded reverse and non-barcoded forward primers were used with Taq DNA polymerase Master Mix (TONBO Biosciences) according to the manufacturer’s directions. Samples were amplified in duplicate with the following thermocycler protocol: hold at 94 °C for 3 min; 30 cycles of 94 °C for 45 s, 50 °C for 1 min, 72 °C for 1.5 min; and hold at 72 °C for 10 min. The duplicate final amplified products were pooled. 16S rRNA gene amplicons were cleaned using Mag-Bind RxnPure Plus beads (Omega Bio-tek). Samples were mixed in equimolar amounts before sequencing on Illumina’s MiSeq platform using 2 × 250 bp paired-end runs. Sequence data are available in the National Center for Biotechnology Information (NCBI) sequence read archive under BioProject PRJNA718322.

Sequence data processing was performed using the QIIME 2 (v.2020.2) pipeline^71^. The DADA2 method^27^ was applied to quality-filter sequences and categorize ASVs. The resulting ASVs were assigned taxonomy by mapping with the SILVA 132 database^72^. The QIIME output data were imported to RStudio (v.1.0.136) with the Bioconductor package phyloseq^73^ for normalizing and plotting the input data. The volcano plot for identifying differentially abundant taxa between the two sorted fractions was analysed and plotted via DESeq2 (ref. ^74^) and EnhancedVolcano^75^ respectively. Fold-enrichment analysis for each ASV in the sorted population was performed according to Ronda et al.^76^. This involved calculating the relative abundance of ASVs in the unsorted population as the normalized number of reads in a sample, and the fold-enrichment of each ASV in the Alk+ and Alk– populations was further calculated as the relative abundance in the sorted population divided by the relative abundance in the unsorted total population.

### Shotgun metagenomic library construction and sequencing

To minimize potential contamination from eukaryote DNA (mice and human), both a host depletion and a microbial DNA enrichment step were conducted before Illumina library preparation with the HostZERO Microbial DNA Kit (Zymo Research). Purified DNA was quantified using the Qubit dsDNA High-Sensitivity Assay Kit (Invitrogen). The sequence libraries were prepared using the NexteraXT DNA Library Preparation Kit (Illumina) followed by size and quality assessment using a Bioanalyzer (Agilent). After quantification with the Qubit 3.0 fluorometer, libraries were pooled and sequenced on an Illumina NextSeq 500 using a paired-end 2 × 150 bp protocol. Sequence data are available in the NCBI sequence read archive under BioProject PRJNA718322.

The raw read data were first processed using Kneaddata^77^ (v.0.6.1). Briefly, this module includes quality trimming (with 4-mer windows with mean Phred quality <25) by Trimmomatic^78^ and host-derived sequence removal by mapping with bowtie2 against the reference genome^79^. For taxonomic classification, Kraken2 was used to assign taxonomy to the Kneaddata-filtered reads^80^, and the resulting assigned taxonomy was further subjected to Bracken2 for estimating the species abundance^81^. The resulting kreport files were converted to MetaPhlAn-compatible file using KrakenTools^80^ for the subsequent establishment of taxonomic and phylogenetic trees using GraPhlAn pipeline^82^. For functional annotation, we applied the Human Microbiome Project Unified Metabolic Analysis Network 3 (HUMAnN3) pipeline that maps reads to functionally annotated organism genomes and uses a translated search to align unmapped reads to UniRef90 protein clusters^83^. Also, to focus on the functional expression of the Alk+ fraction of interest, the gene family clusters were determined using Welch’s two-sided *t*-test followed by Benjamin–Hochberg FDR correction (Supplementary Table 4). Only clusters identified as significantly abundant in the Alk+ fraction were retained for plotting. The unstratified gene family abundances were further converted to Gene Ontology terms for assessing the general metabolic activities^84^. The detailed HUMAnN workflow is published in Franzosa et al.^85^.

### Heterologous expression and in vivo activity of cholesterol sulfotransferase candidates

Cholesterol sulfotransferase candidates were selected from BLAST analysis of the *B. thetaiotaomicron* genome with the amino acid sequences of human SULT2A1 and SULT2B1. The six candidates were then amplified from the genome of *B. thetaiotaomicron* with the primers listed in Supplementary Table 1 and cloned into pET28a *E. coli* expression vector with the NdeI and BlpI cut sites. Ligated plasmid was transformed into TOP10 *E. coli* and screened via colony PCR for positive insertions. Positive pET28 sulfotransferase plasmids were purified with the QIAPrep Spin Miniprep Kit (Qiagen) and were verified via Sanger sequencing. Confirmed plasmids were transformed into BL21 *E. coli* (New England Biolabs) and selected with Luria broth (LB) plates containing 50 μg ml^−1^ kanamycin. An individual colony of each pET28 sulfotransferase transformed candidate was picked into separate 5 ml of LB media and cultured overnight at 37 °C in 15 ml plastic culture tubes with shaking at 250 r.p.m. Some 50 μl of the overnight cultures were used to inoculate 6 ml of M9 media containing glucose treated with either cholesterol or Chol^Alk^. No isopropylthiogalactoside was needed because expression from the T7 promoter is leaky. After shaking at 37 °C 250 r.p.m. for 18 h, cells were centrifuged at 4,000*g* for 20 min at 4 °C and the spent media was removed. The bacterial pellets were resuspended in 1 ml of PBS and moved to 1.7 ml centrifuge tubes. Samples were centrifuged again at 4,000*g* and 4 °C for 10 min and the PBS removed. Samples were frozen with liquid nitrogen, lyophilized to dryness and underwent metabolite extraction and LC–MS analysis as described below.

### Immobilized metal-affinity chromatography enrichment and in vitro activity of polyhistidine-tagged BT_0416 from *E. coli*

BL21 *E. coli* containing pET28 *Bt_0416* plasmid was grown in 10 ml of LB overnight. The 10 ml of overnight culture was then diluted into 1 l of terrific broth containing kanamycin and cultured at 37 °C in a 2 l Erlenmeyer flask rotating at 250 r.p.m. Once an optical density at 600 nm (OD_600_) of 0.6 was reached, culture temperature was reduced to 20 °C, isopropylthiogalactoside was added to a final concentration of 100 μM and cultured for an additional 16 h. Cells were harvested at 4,000*g* for 12 min at 4 °C and resuspended in 80 ml of lysis buffer (20 mM sodium phosphate pH 7.4, 300 mM sodium chloride, 1× ProBlock Gold Bacteria 2D Protease Inhibitor Cocktail; Gold Biotechnology). The slurry was sonicated with a probe sonicator and then centrifuged again at 20,000*g* for 20 min at 4 °C. The supernatant was applied to pre-equilibrated Ni-NTA (Gold Biotechnology). The lysate was gravity fed through the beads and the beads were washed with 15 ml of lysis buffer. The captured protein was eluted with 15 ml of lysis buffer containing 250 ml of imidazole and 10% glycerol. The eluent was concentrated to 500 μl with an Amicon Ultra-15 30 K distinct molecular weight cutoff spin filter (Merck Millipore) and flash-frozen over liquid nitrogen until further analysis. Some 5 μl of partially isolated BT_0416 (Supplementary Fig. 8) was added to a 100 μl scale reaction solution containing 50 mM potassium phosphate pH 7.5, 250 μM cholesterol, 250 μM PAPS (Sigma-Aldrich) and 0.2% Triton X-100 and incubated at 37 °C for 12 h. Solutions were dried with a SpeedVac Vacuum Concentrator. Samples then underwent metabolite extraction and LC–MS analysis as described below.

### BT_0416 knockout strain (*BtΔtdkΔ0416*)

Mutagenesis was performed as described in Johnson et al.^86^. Specifically, to generate an in-frame deletion of *Bt_0416* in *B. thetaiotaomicron*, the strain *B. thetaiotaomicron* VPI-5482 *tdk* was used^87^. Two 700 bp regions, each flanking the gene to be deleted, were PCR-amplified (NEB Q5 Hot Start High-Fidelity DNA Polymerase) and cloned into EcoRV-HF and NotI-HF linearized pExchange_tdk. Primers are listed in Supplementary Table 1 and strains are listed in Supplementary Table 2. The assembled construct was transformed into *E. coli* S17-1 λpir (Biomedal), plated on LB agar–streptomycin–carbenicillin plates, and transformants screened for incorporation of the plasmid. Final concentrations of antibiotics and selection agents were as follows: erythromycin 25 μg ml^−1^, gentamicin 200 μg ml^−1^, streptomycin 100 μg ml^−1^, carbenicillin 100 μg ml^−1^, 5′-fluoroxyuridine (FUdR) 200 μg ml^−1^. To conjugate cells, recipient and donor cells were inoculated from overnight cultures (*B. thetaiotaomicron tdk* at 1:1,000; *E. coli* transformant at 1:250) and grown to early exponential phase (OD_600_ = 0.2–0.3), at which time the donor and recipient strains were combined in a 1:1 ratio and centrifuged for 20 min at 3,220*g* at room temperature. The bacterial pellet was resuspended in 100 μl of brain heart infusion broth-supplemented with 5 g l^−1^ yeast extract and 5 μg ml^−1^ hemin (BHIS), plated as a puddle on BHIS–10% defibrinated sheep blood agar plates, and incubated anaerobically at 37 °C for 20 h. The conjugation puddle was then scraped, serially diluted in PBS and incubated aerobically at 37 °C on BHIS–10% defibrinated sheep blood–gentamicin–erythromycin agar plates. Colonies were screened for merodiploids via PCR, cultured overnight in liquid BHIS and serially diluted onto BHIS–10% defibrinated sheep blood–gentamicin–FUdR agar plates. Colonies were PCR screened for deletion of the gene and confirmed via Sanger sequencing and metabolomics.

### Phylogenetic tree of *Bt_0416*-like genes in various organisms

A BLASTP analysis was performed utilizing the BLAST tool from the Kyoto Encyclopedia of Genes and Genomes (https://www.kegg.jp/) tool set. The top 50 results were selected and the TREE function was applied. Alignment and phylogenetic reconstructions were performed using the function ‘build’ of ETE3 v.3.1.1 (ref. ^88^). Alignment was performed with MAFFT v.6.861b using the default options^89^. The tree was constructed using FastTree v.2.1.8 with default parameters^90^.

### Monocolonization and conventionalization of GF mice

Forty 5-week-old female GF Swiss Webster mice were purchased from Taconic Biosciences. Mice were randomly assigned to one of the four groups: (1) GF; (2) WT, *BtΔtdk* mono-associated; (3) KO, *BtΔtdkΔ0416* mono-associated; and (4) CONV-D, conventionalized (three cages per group, and three or four mice per cage). For monocolonization experiments, colonization was achieved by a single oral gavage with 10^9^ c.f.u. of either *BtΔtdk* (cholesterol sulfotransferase competent) or *BtΔtdkΔ0416* (cholesterol sulfotransferase null) in 200 μl of sterile PBS. Mice were maintained monocolonized in sterile cages on a Rodent NIH-31 Modified Diet (Zeigler) and were given access to sterile autoclaved water ad libitum. For conventionalization, 1 g of faecal pellets were freshly collected from specific pathogen-free Swiss Webster mice (Taconic Biosciences) and resuspended in 15 ml of sterile PBS supplemented with 0.1% (w/v) l-cysteine hydrochloride, the faecal slurry then sat at 37 °C anaerobically for 5 min, the resulting supernatant was collected and 200 μl of the suspension per mouse was introduced to a GF mouse via oral gavage. Two weeks post-colonization, faecal pellets were collected, and mice were euthanized by decapitation. Blood was collected, with 300 μl of blood mixed with 250 µg ml^−1^ freeze-dried heparin for the whole-blood sample, and the rest was equilibrated at room temperature for 1.5 h, followed by centrifugation at 3,000 r.p.m. for 15 min to retrieve serum samples. Caecal content was collected, snap-frozen in liquid nitrogen, and stored at −80 °C.

### Evaluation of *Bacteroides* colonization

Faecal pellets from each group of mice were weighed, mashed and vortexed in 1 ml of sterile PBS, and diluted to plate c.f.u. on triplicate Brain Heart Infusion agar plates supplemented with 10% defibrinated horse blood. Plates were placed in a 37 °C incubator inside an anaerobic chamber (Coy Laboratory Products). Plates were removed after 48 h and colonies were counted manually.

### Evaluation of bacterial load

Bacterial load was determined by real-time qPCR using a protocol modified from Gomes-Neto et al.^91^. In brief, an aliquot of caecal content was resuspended with 750 μl of lysis buffer (200 mM NaCl, 100 mM Tris pH 8.0, 20 mM EDTA, 20 mg ml^−1^ lysozyme), and transferred to a sterile screw-top tube containing ~500 mg of 0.1 mm zirconium beads (BioSpec Products). After extraction at 350 r.p.m. and 37 °C for 30 min, 85 μl of 10% SDS solution and 40 μl of proteinase K (15 mg ml^−1^, Qiagen) were added, and the samples incubated for 10 min at 55 °C. Samples were then homogenized in a Mini-BeadBeater (BioSpec) for 1 min at maximum speed. After cooling on ice for 3 min, 500 μl of phenol–chloroform–isoamyl alcohol (25:24:1, Sigma-Aldrich) were added, and tubes were inverted ten times until there was more or less no layer separation, followed by centrifugation at 18,000*g* for 5 min. The aqueous layer was transferred to a new sterile microcentrifuge tube containg the same volume of chloroform–isoamyl alcohol (24:1, Sigma-Aldrich). After centrifugation at 18,000*g* for 5 min, the final aqueous layer was transferred to a new sterile microcentrifuge tube and 0.6 vol. of ice-cold isopropanol was added. After overnight precipitation at −20 °C, DNA was recovered by centrifugation at 18,000*g* for 30 min at 4 °C, followed by three rounds of 75% nuclease-free ethanol washing. The final DNA pellet was resuspended in 100 μl of nuclease-free water. Bacterial DNA was amplified with universal 16S primers as listed in Supplementary Table 1 using Power SYBR Green PCR Master Mix (Thermo Fisher Scientific) and QuantStudio 7 real-time PCR system (Applied Biosystems). The results were normalized to caecal content weight.

### Metabolome extraction

One millilitre of methanol was added to the dried material and sonicated for 3 min, with on/off cycles of 3 s on, 2 s off, at 100% power on a Qsonica Ultrasonic Processor with the Cup Horn water bath attachment maintained at 20 °C. The samples were then placed on an end-over-end rotator and metabolites were extracted overnight. Samples were then centrifuged at 18,000*g* at 4 °C for 30 min. The clarified supernatant was collected and transferred to a fresh 1.7 ml centrifuge tube. The collected extracts were evaporated to dryness using a SpeedVac vacuum concentrator (Thermo Fisher Scientific) and reconstituted in 200 µl of methanol. Samples were sonicated again and centrifuged at 18,000*g* at 4 °C for 30 min. In total, 150 µl of clarified concentrated extracted metabolome was transferred to an HPLC vial utilizing an insert (Thermo Fisher Scientific) and stored at 4 °C until LC–MS analysis.

### LC–MS analysis for untargeted metabolomics

High-resolution LC–MS analysis was performed on a Thermo Fisher Scientific Vanquish Horizon UHPLC System coupled with a Thermo Q Exactive HF hybrid quadrupole-orbitrap high-resolution mass spectrometer equipped with a heated electrospray ionization (HESI) ion source. Five microlitres of concentrated extract was injected and separated using a water–acetonitrile gradient on an Agilent Technologies InfinityLab Poroshell 120 EC-C18 column (50 mm × 2.1 mm, particle size 2.7 μm, part no. 699775-902) maintained at 50 °C. Solvent A was 0.1% formic acid in water and solvent B was 0.1% formic acid in acetonitrile. The A/B gradient started at 20% solvent B for 1 min after injection, increased linearly to 100% solvent B at 16 min and was held at 100% solvent B for 5 min, at a flow rate 0.6 ml min^−1^. Mass spectrometer parameters were as follows: spray voltage, 3.5 kV (positive mode) and 3.0 kV (negative mode); capillary temperature, 380 °C; prober heater temperature, 400 °C; sheath, auxiliary and spare gas of 60, 20 and 2, respectively; S-lens RF level 50, resolution 240,000 at *m*/*z* 200, AGC target 3 × 10^6^. Each sample was analysed in positive and negative modes with an *m*/*z* range of 150–800.

### Untargeted metabolomic analysis

Untargeted metabolomic analysis RAW files generated from HPLC–HRMS acquisitions were converted to mzXML files utilizing MSconvertGUI software (proteowizard.sourceforge.net)^92^. Differential molecular features were determined by subjecting mzXML files to Metaboseek Software v.0.9.6 (metaboseek.com) utilizing the XCMS package^42,44^. Differential features were filtered using the minFoldOverCtrl, minInt and Fast_Peak_Quality filters, and then curated manually to remove adducts and isotopes (Supplementary Tables 5 and6). The curated features were assigned molecular formulas and then subjected to MS/MS. The MS/MS fragments were also assigned molecular formulas and structures were inferred. A focus was placed on structures that could be inferred.

Metabolomes were compared between caecal contents of mice orally exposed to Chol^Alk^, cholesterol or vehicle control (olive oil gavage) in the in vivo condition (Supplementary Table 5). In the ex vivo condition, metabolomes were compared between faecal cultures grown in multiple media conditions (media A, media B), these conditions plus ethanol as a vehicle control (media A + ethanol, media B + ethanol), these conditions supplemented with cholesterol (media A + cholesterol, media B + cholesterol) and these media conditions supplemented with Chol^Alk^ (media A + Chol^Alk^, media B + Chol^Alk^) (Supplementary Table 6).

### LC–MS analysis for targeted metabolomics

Targeted LC–MS analysis was performed on a Thermo Scientific Vanquish Horizon UHPLC System coupled with a Thermo Scientific TSQ Quantis Triple Quadrupole mass spectrometer. Analytes were separated using an Agilent Technologies InfinityLab Poroshell 120 EC-C18 column (50 mm × 2.1 mm, particle size 2.7 μm, part no. 699775-902) maintained at 50 °C. The mass spectrometer was calibrated using Pierce Triple Quadrupole Calibration Solution Extended Mass Range solution. For MS/MS acquisition: source fragmentation 0 V, collision energy 30 V, CID gas 1.5 mTorr (argon).

Positive mode analysis was performed with an atmospheric pressure chemical ionization (APCI) ion source. Mobile phase A was 99.9% water with 0.1% formic acid (v/v). Mobile phase B was 99.9% acetonitrile with 0.1% formic acid. The A/B gradient started at 20% B for 1 min after injection and increased linearly to 100% B at 5 min, held at 100% B for 10 min, using a flow rate 0.6 ml min^−1^. Full Scan Q1 mass spectrometer parameters were as follows: spray current, static; positive ion discharge current, 4; negative ion discharge current, 10; ion transfer tube temperature, 275 °C; vaporizer temperature 35 °C; sheath, auxiliary and spare gas 45, 5 and 1, respectively. Samples were analysed with an *m*/*z* range of 200–1,000.

Negative mode analysis was performed with a HESI ion source. Mobile phase A was 94.9% water, 5% methanol and 0.1% formic acid (v/v) containing 10 mM ammonium acetate. Mobile phase B was 99.9% methanol with 0.1% formic acid. A/B gradient started at 15% B for 1 min after injection, increased linearly to 100% B at 20 min and was held at 100% B for 4 min, using a flow rate 0.6 ml min^−1^. Full Scan Q1 mass spectrometer parameters were: spray voltage, 2.5 kV in positive mode; ion transfer tube temperature, 350 °C; vaporizer temperature, 350 °C; sheath, auxiliary and spare gas 60, 15 and 2, respectively. Samples were analysed with an *m*/*z* range of 200–1,000.

Chol^Alk-Sulf^ in hepatic portal vein blood was monitored with selective reaction monitoring with mass transitions: *m*/*z* 465.3→96.94 and collision energy at 30 V.

### RT–qPCR analysis of *Bt_0412* and *Bt_0416*

*B. thetaiotaomicron* (*Bt*) was maintained in BHIS media. One millilitre of stationary phase *Bt* was diluted in 25 ml of 10% tryptone medium and cultured as described in Bjursell et al.^51^. After 6 h, the log phase culture was diluted again (1 ml in 25 ml) in fresh tryptone medium containing either vehicle, lactose, cholesterol or lactose and cholesterol. For cholesterol-containing experiments, cholesterol was added to a final concentration of 10 μM. The cultures were harvested after 3 h incubation via centrifugation at 4,000*g* for 20 min. After removing the supernatant, bacterial pellets were resuspended in 125 μl of PBS and 150 μl of TRIzol reagent (Thermo Fisher Scientific), along with 0.5 mm zirconium beads, homogenized on a Mini-BeadBeater for 1 min and then moved to ice for 3 min. Thirty microlitres of chloroform was added and lightly inverted. Samples were centrifuged at 18,000*g* at 4 °C for 20 min. Then 300 μl of the RNA-containing fraction was applied to the RNA Clean & Concentrator-5 (Zymo Research) kit and RNA purified as per the manufacturer’s protocol. Some 20 μg of RNA was then reverse transcribed via the High-Capacity cDNA Reverse Transcription Kit (Applied Biosystems) as per the manufacturer’s protocol utilizing the random primers supplied with the kit. RT–qPCR was carried out using the complementary DNA to Power SYBR Green PCR Master Mix (Thermo Fisher Scientific) with the qPCR primers listed in Supplementary Table 1 on a QuantStudio 7 Real-time PCR system (Applied Biosystems). All transcript quantifications were normalized to the 16S rRNA gene quantification.

### Bacterial growth assay in cholesterol-supplemented media

Bacteria were maintained in BHIS broth. Cultures were allowed to reach stationary phase in BHIS and subsequently incubated at 22 °C for 24 h before use. Cholesterol minimal media was prepared by adding 1% Tween 80 (v/v) to the media and adding cholesterol to saturation. Excess cholesterol was removed via centrifugation, and a cholesterol dilution series was established. Cholesterol concentrations were determined via LC–MS. Input bacteria was normalized, inoculated into the various media supplemented with cholesterol, and cultured at 37 °C in an anaerobic chamber in a 96-well plate. The OD_600_ values of the bacterial cultures were read using a BioTek Epoch 2 microplate spectrophotometer at 6 and 30 h.

### Evaluation of transfer of labelled bacterial Chol^Sulf^ to mouse hepatic portal vein

To specifically track the transfer of bacterial Chol^Sulf^, 1 ml of *BtΔtdk* (cholesterol sulfotransferase competent) or *BtΔtdkΔ0416* (cholesterol sulfotransferase null) was inoculated into 25 ml of prewarmed (37 °C) 25 μM Chol^Alk^ supplemented minimal media^86,93^ consisting of 13.6 g of KH_2_PO_4_, 0.875 g of NaCl, 1.125 g of (NH_4_)_2_SO_4_, 5 g of glucose, (pH to 7.2 with concentrated NaOH), 1 ml of hemin solution (500 mg dissolved in 10 ml of 1 M NaOH, then diluted to final volume of 500 ml with water), 1 ml of MgCl_2_ (0.1 M in water), 1 ml of FeSO_4_⋅7H_2_O (1 mg per 10 ml of water), 1 ml of vitamin K3 (1 mg ml^−1^ in absolute ethanol), 1 ml of CaCl_2_ (0.8% w/v), 250 μl of vitamin B12 solution (0.02 mg ml^−1^) and 5 g of l-cysteine hydrochloride anhydrous). After incubation overnight at 37 °C, bacterial pellets were harvested by centrifugation at 4,000*g* for 20 min at room temperature. The cells were pelleted and washed twice with filtered sterile 0.1% BSA/ PBS to remove residual Chol^Alk^ and the resulting bacterial cells were resuspended in sterile PBS. The gavage amount of *BtΔtdk*(Chol^Alk^) or *BtΔtdkΔ0416*(Chol^Alk^) was adjusted to an OD_600_ of ~1.0 per 200 μl of PBS. One millilitre of *BtΔtdk*(Chol^Alk^) and *BtΔtdkΔ0416*(Chol^Alk^) were frozen by liquid nitrogen and dried via lyophilization for verifying the presence of Chol^Alk-Sulf^.

After 4 d of acclimation in a climate-controlled environment with 12 h light and dark cycles, nine 6-week-old Swiss Webster mice (Taconic Biosciences) were randomly assigned to two groups and orally gavaged once with either *BtΔtdk*(Chol^Alk^) or *BtΔtdkΔ0416*(Chol^Alk^). Mice were reared on a standard sterilized breeder diet (LabDiet 5021) with ad libitum access to autoclaved water. After 2 h, mice were euthanized by CO_2_ followed by cervical dislocation. Some 30 IU ml^−1^ of heparin was added to the body cavity around the hepatic portal vein before collecting the blood samples by Pasteur pipette^86^. Hepatic portal vein blood was then frozen by liquid nitrogen and dried via lyophilization for metabolome extraction.

### Human stool metabolomics

Human stool samples for testing the ability of human gut microbes to produce Chol^Sulf^ were analysed as subset of a study measuring stool metabolites during the first 3 months of life. The study protocol was approved by the Cornell Institutional Review Board for Human Subjects Research (protocol no. 2007009697) and all participants provided written informed consent. For infant participants, consent was provided by their immediate caregiver. Study incentives included a 3-month supply of nappies. Two stool samples collected from infant diapers (male, 0–3 months of age) were stored at −20 °C until thawed for use in ex vivo cultures as described above (Ex vivo culture of live faecal microbes with cholesterol or Chol^Alk^).

### Statistical analysis

No statistical methods were used to predetermine sample sizes but our sample sizes are similar to those reported in previous publications^55,93^. Statistical tests were performed using GraphPad Prism 9 or in R. No animals or data points were excluded from the analysis. Data distribution was assumed to be normal but this was not formally tested. Owing to the nature of the treatment conditions, data collection and analysis were not performed blind to the conditions of the experiments. Statistical tests are denoted in the figure legends and Methods.

### Reporting summary

Further information on research design is available in the Nature Research Reporting Summary linked to this article.

## Supplementary information

Supplementary Information Supplementary Figs. 1–9 and Tables 1–3.

Reporting Summary

Supplementary Tables Supplementary Table 4: Metagenomic sequencing data. Supplementary Table 5: LC–MS data for in vivo work. Supplementary Table 6: LC–MS data for in vivo and ex vivo work.

## Data Availability

Metagenomic and V4-16S rRNA gene sequencing data are deposited as fastq files filed in NCBI Sequence Read Archive under BioProject PRJNA718322. Metabolomics LC–MS data has been deposited at MassIVE with the accession number MSV000087688. Source data are provided with this paper.

## Source data

Source Data Fig. 4 Statistical source data.
Source Data Extended Data Fig. 8 Statistical source data.
Source Data Extended Data Fig. 9 Statistical source data.

## Code availability

No custom code was used for this study.
