# Supplementary material for: Characterization of interactions of dietary cholesterol with the murine and human gut microbiome
Source: Nat Microbiol. 2022 Aug 18;7(9):1390–403. doi: 10.1038/s41564-022-01195-9 (PMC9417993; doi:10.1038/s41564-022-01195-9)
Supplement: Supplementary file 1 — Supplementary Figs. 1–9 and Tables 1–3. [file 41564_2022_1195_MOESM1_ESM.pdf]

nature portfolio

https://doi.org/10.1038/s41564-022-01195-9

Supplementary information

Characterization of interactions of dietary cholesterol with the murine and human gut microbiome

In the format provided by the authors and unedited

Supplementary Information

Characterization of interactions of dietary cholesterol with the murine and human gut microbiome

Henry H. Le[1*], Min-Ting Lee[1*], Kevin Besler[1], Janine Comrie[1], Elizabeth L. Johnson[1,2]

[1]Division of Nutritional Sciences, Cornell University, Ithaca, NY 14853, USA
[2]Correspondence to: elj54@cornell.edu
*These authors contributed equally to this work

Supplemental Tables

Supplementary Table 1 - Primers

Name/Use	Sequence
pET28_BT0416_fwd	gtgccgcgcggcagccatatgGGATTACTTGAATTTAATAAACTTCCGATTAATACGTTG
pET28_BT0416_rev	gttatgctagttattgctcagcTTATAAATTATAGTTCCATTGTTTCAAAGCAAAATCCC
pET28_BT0468_fwd	ggtgccgcgcggcagccatatgATAAAAATAAAAAAATCAGAGGATTGTTGTGGATGTTC
pET28_BT0468_rev	ctagttattgctcagcTTATTGGAGTTTTCTTAATACTCTTTTAATAATTTGTTTCACTA
pET28_BT0487_fwd	gcggcctggtgccgcgcggcagccatatgAATAAATTGCCCGAAAGAATCAGAATCAAAG
pET28_BT0487_rev	ggttatgctagttattgctcagcTTATTTGCTATGGTAATAGTCTATTGTTTCTACAGTC
pET28_BT0969_fwd	gcctggtgccgcgcggcagccatatgAATAATCTTCGAATCTTATTATACGTGGTTGTCC
pET28_BT0969_rev	ccccaaggggttatgctagttattgctcagcCTACAATGATTTGATGTAGTCTTCCAGTG
pET28_BT1511_fwd	ggtgccgcgcggcagccatatgAAAATAAGAAATTTATTAGTAGCCATACTTGCAATGAG
pET28_BT1511_rev	aaggggttatgctagttattgctcagcTCATTCTGCTATACAGATACAAACACGATTTTC
pET28_BT1712_fwd	agcggcctggtgccgcgcggcagccatatgAAAAAGGTAGATATAATTCATGGTGGATGG
pET28_BT1712_rev	gttatgctagttattgctcagcTCATATAGCGTTCAAAATATTAGCATAAGATTCAATCA
pEx_frag1_0416_fwd	CGAGTCGACGGTATCGATAAGCTTGATATCAAGTGGCATCGGGTATTGTCCGCAAGGGAG
frag1_0416_fwd	AAGTGGCATCGGGTATTGTCCGCAAGGGAGATACAGTGAT
frag1_0416_rev	TTTCTTAATCATCAATACTTATCCTTTCTTCGTTCTACATTTTATTTAATCACTTTTATG
frag2_0416_fwd	CATAAAAGTGATTAAATAAAATGTAGAACGAAGAAAGGATAAGTATTGATGATTAAGAAA
frag2_0416_rev	AAGTCACCAACGTTCCGATACGCTTGGCATAAACATTTCC
pEx_frag2_0416_rev	GGAATTCCCCTCCACCGCGGTGGCGGCCGCAAGTCACCAACGTTCCGATACGCTTGGCAT
qPCR_BT0416_fwd	CCAGCCTCTGAAACTGGAGG
qPCR_BT0416_rev	TTCTCCGTCATTCCCATCGC
qPCR_BT0412_fwd	CCGTTTCCCCGGTATCAACA
qPCR_BT0412_rev	CGTATCATCCGCCATCACCA
16s_qPCR_fwd	GGTAGTCCACACAGTAAACGATGAA
16s_qPCR_rev	CCCGTCAAATTCCTTTGAGTTTC
515F	AATGATACGGCGACCACCGAGATCTACACTATGGTAATTGTGTGCCAGCMGCCGCGGTAA
806R	CAAGCAGAAGACGGCATACGAGATXXXXXXXXXXXXAGTCAGTCAGCCGGACTACHVGGGTWTCTAAT

Supplementary Table 2 - Strain sources

Strain	Source
A. caccae	DSM 14662
B. caccae	ATCC 43185
B. ovatus	ATCC 8483
B. fragilis	ATCC 25285
B. uniformis	ATCC 8492
B. vulgatus	ATCC 8482
B. thetaiotaomicron	DSM 2079
B. thetaiotaomicron (sphingolipid-deficient)	Professor Andrew Goodman, Yale University
B. thetaiotaomicron VPI-5482 *tdk*	Professor Andrew Goodman, Yale University
B. thetaiotaomicron VPI-5482 *tdk Δ0416*	This study
B. longum subsp. *infantis*	ATCC 15697
B. pseudolongum subsp. *pseudolongum*	ATCC 25526
E. coli (BL21)	New England Biolabs C2530H
E. coli (TOP10)	Invitrogen C404010
E. coli (S17-1 λpir)	Biomedal
E. coprostanoligenes	ATCC 51222
L. amylovorus Nakamura	ATCC 33620
Ruminococcaceae species	ATCC TSD-27
American Type Culture Collection	ATCC
German Collection of Microorganisms	DSM

All unique plasmids and strains available upon request.

Supplementary Table 3 – Sort efficiency

Replicate	# of Alk+ events collected	# of Alk- events collected	% of parent population[a] in Alk+ gate	% of parent population[a] in Alk- gate	% Sort Efficiency in Alk+ gate	% Sort Efficiency in Alk- gate
Mouse_1	1,152,000	1,152,000	36.17%	63.70%	95%	94%
Mouse_2	1,152,000	1,152,000	40.38%	59.42%	93%	93%
Mouse_3	1,152,000	1,152,000	34.87%	65.12%	98%	97%
exvivo_1	1,103,000	1,103,000	37.61%	62.37%	95%	96%
exvivo_2	1,152,000	1,152,000	36.12%	63.27%	96%	96%
exvivo_3	1,018,000	1,023,010	38.12%	60.33%	98%	97%

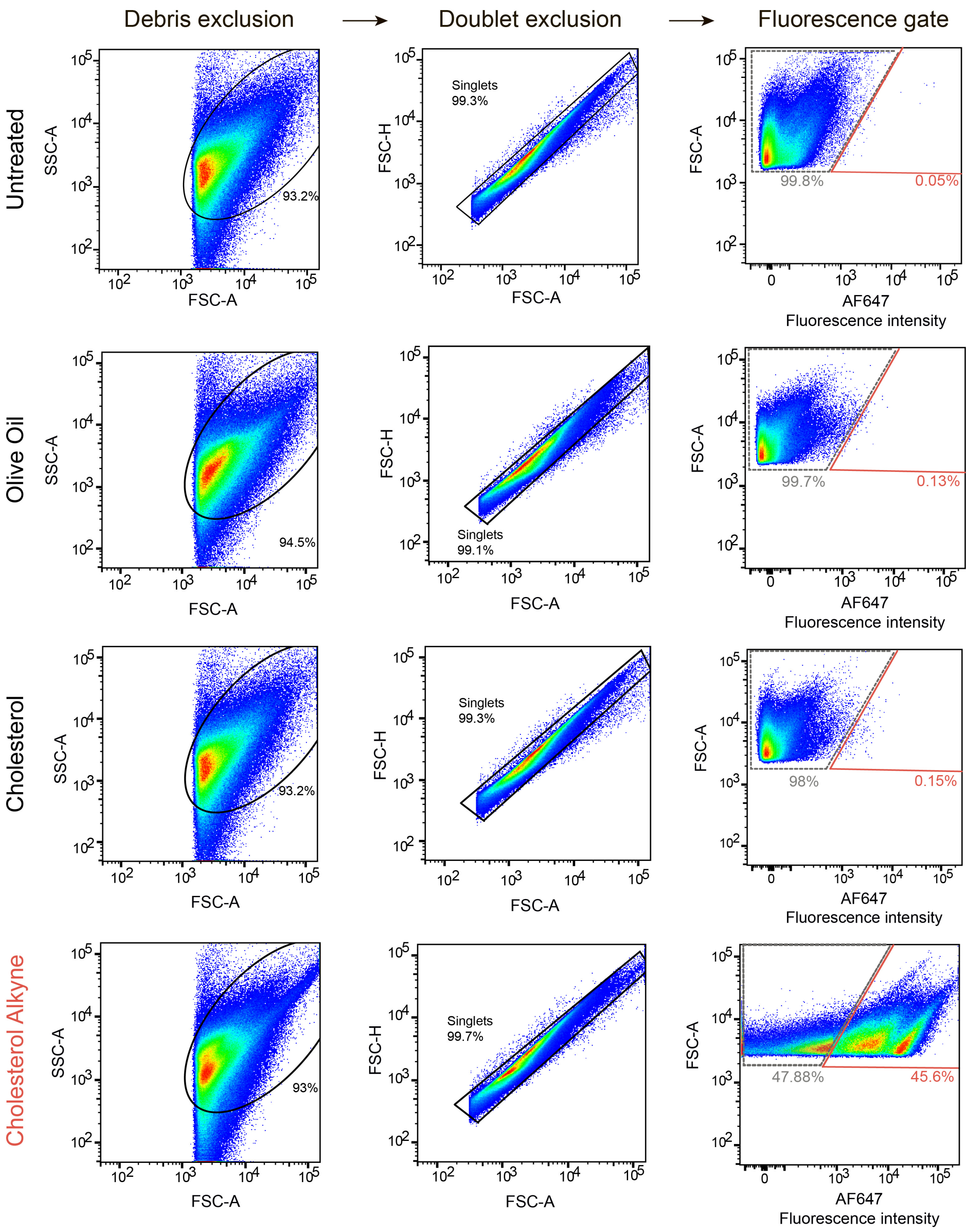

Supplementary Fig. 1| Representative density plots from successive FACS gating steps applied to cecal microbial samples from each dietary treatment group. This gating strategy demonstrates the steps used to isolate Alk+ and Alk- populations. The definition of each step is outlined on the top of the panel, and the treatment group is shown on the left side of each row.

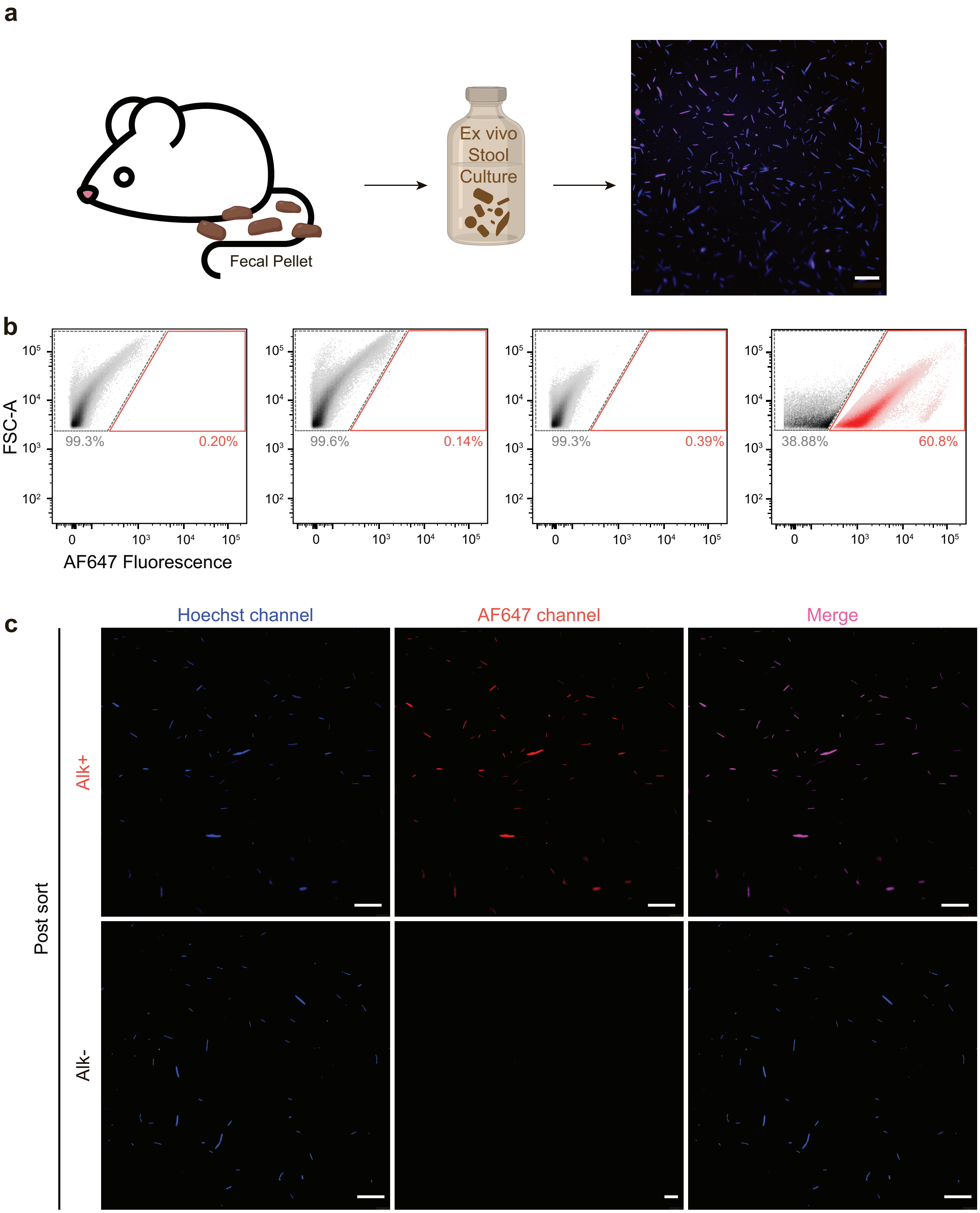

Supplementary Fig. 2 | FACS enrichment of AF647-azide positive microbes arising from *ex vivo* culturing system. a, Fecal microbes were extracted from murine fecal pellets and cultured with Chol^{Alk}. Bacterial uptake was confirmed by fluorescent microscopy and the merged image is shown. Chol^{Alk}-interacting microbes are red (AF647-azide) while the nucleic acids stain marks all microbes in blue using Hoechst 33342. (Scale bar: 20 µm) **b**, FACS density plots

demonstrating isolation of Chol^{Alk}-interacting organisms. **c**, Fluorescence microscopy demonstrating staining of Chol^{Alk}-interacting or non-interacting organisms post-FACS. (Scale bar: 20 µm).

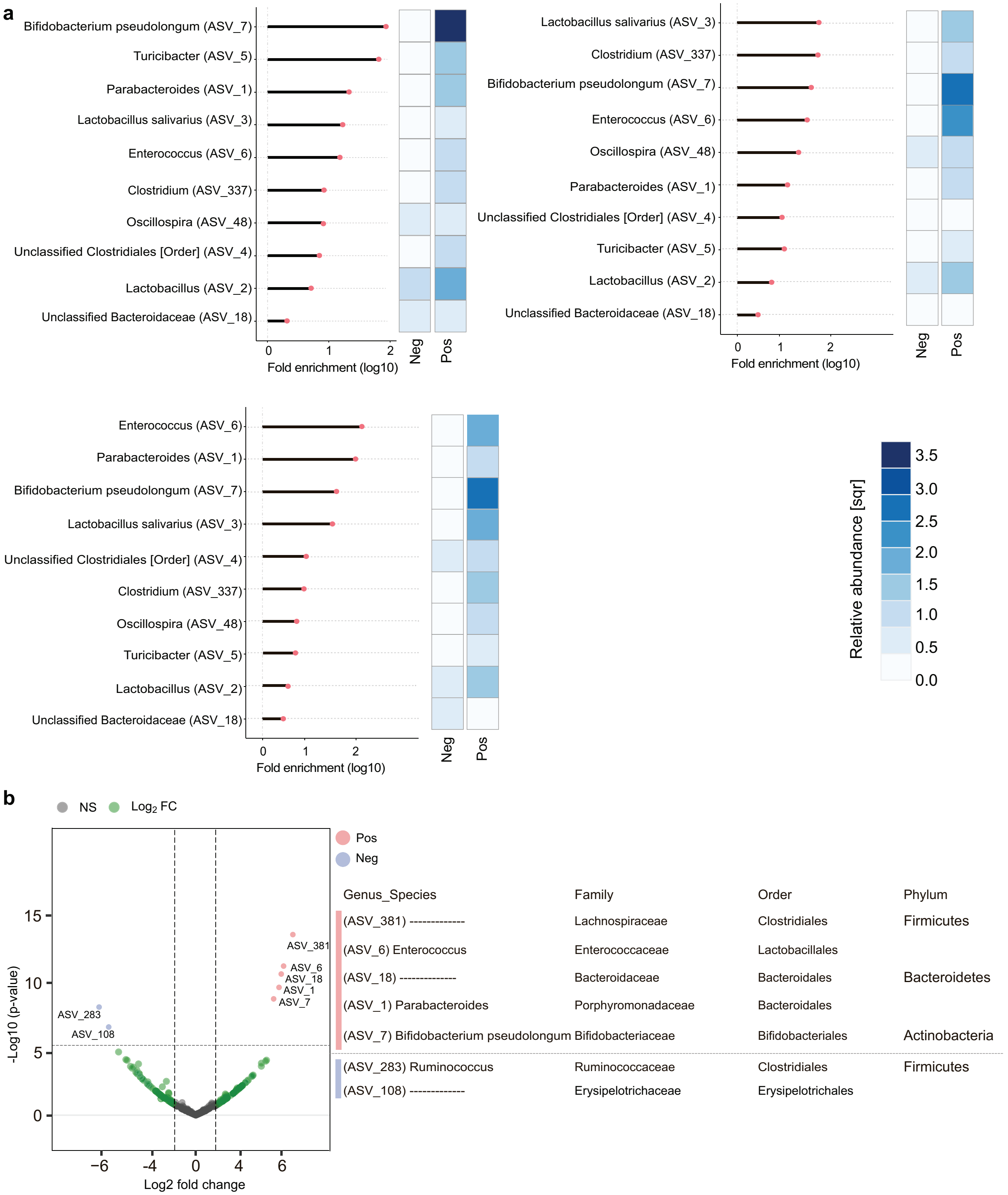

Supplementary Fig. 3 | BioOrthogonal-labeling Sort-Sequence- (BOSS-) reveals the taxonomic identities of cholesterol-interacting gut microbes. a, Enrichment results from 16S amplicon sequencing determined taxonomic classifications of cholesterol-interacting microbes separated using FACS. Heat maps indicate the magnitude of representation of the listed taxa in the neg (Alk-, cholesterol non-interacting) and pos (Alk+, cholesterol-interacting) sorted fractions. (n = 3 for each sorted population). Red dots indicate the value of the fold enrichment. **b**, Volcano plot showing taxonomic differences between Alk+ and Alk- using DESeq2 analysis. The pink dots represent those ASVs that have a $\log_2$ fold change greater than 2 enriched in Alk+ and are significantly different (Wald test, Benjamin Hochberg correction, adjusted $P < 0.05$) from the Alk- fraction. (n = 3 for each sorted population, Pos: Alk+ and Neg: Alk-).

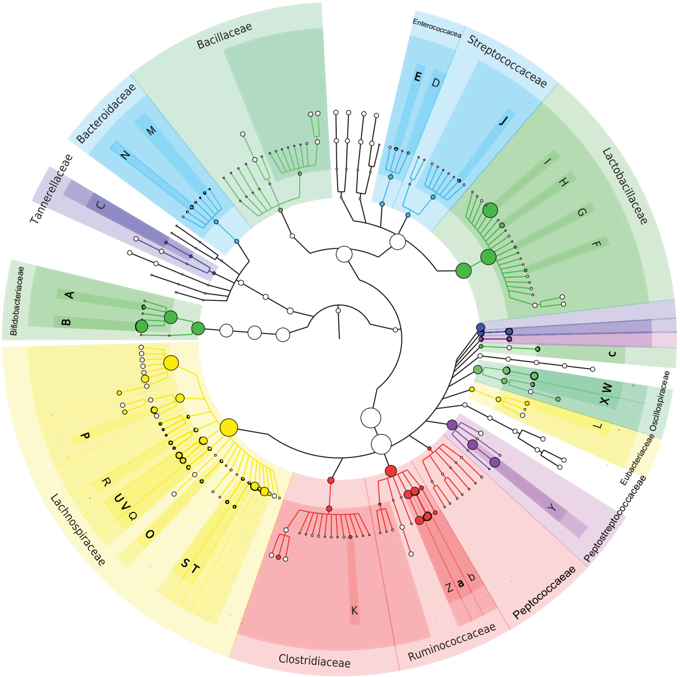

Bifidobacteriaceae	A: Bifidobacterium bifidum<br>B: Bifidobacterium pseudologum
Tannerellaceae	C: Parabacteroides johnsonii
Enterococcaceae	D: Enterococcus cecorum<br>E: Enterococcus faecalis
Lactobacillaceae	F: Lactobacillus amylovorus<br>G: Lactobacillus salivarius<br>H: Lactobacillus rhamnosus<br>I: Lactobacillus murinus
Streptococcaceae	J: Streptococcus dysgalactiae
Clostridiaceae	K: Clostridium scidens
Eubacteriaceae	L: Eubacterium maltosivorans
Bacteroidaceae	M: Bacteroides fragilis<br>N: Bacteroides ovatus
Lachnospiraceae	O: Anaerostipes caccae<br>P: Anaerostipes hardrus<br>Q: Anaerostipes rhamnosivorans<br>R: Blautia producta<br>S: Lachnospiraceae bacterium<br>T: Pseudobutyrivibrio xylanivorans<br>U: Roseburia inulinivorans<br>V: Roseburia intestinalis
Oscillospiraceae	W: Dysosmobacter welbionis<br>X: Oscillibacter valericigenes
Peptostreptococcaceae	Y: Clostridioides difficile
Ruminococcaceae	Z: Faecalibacterium prausnitzii<br>a: Flavonifractor plautii<br>b: Ruminococcus gnavus
Flincbacter	c: Flintibacter sp. KGMB00164

Supplementary Fig. 4 | Shotgun metagenomic sequencing analysis identifies various taxa enriched in the Alk+ fraction. Each dot represents a node in the phylogenetic tree, and its size indicates the prevalence of representatives of the corresponding node in the sample. Family-level taxonomic classification is colored for differentiation. The letters represent those microbes identified with relatively high abundance with species-level resolution.

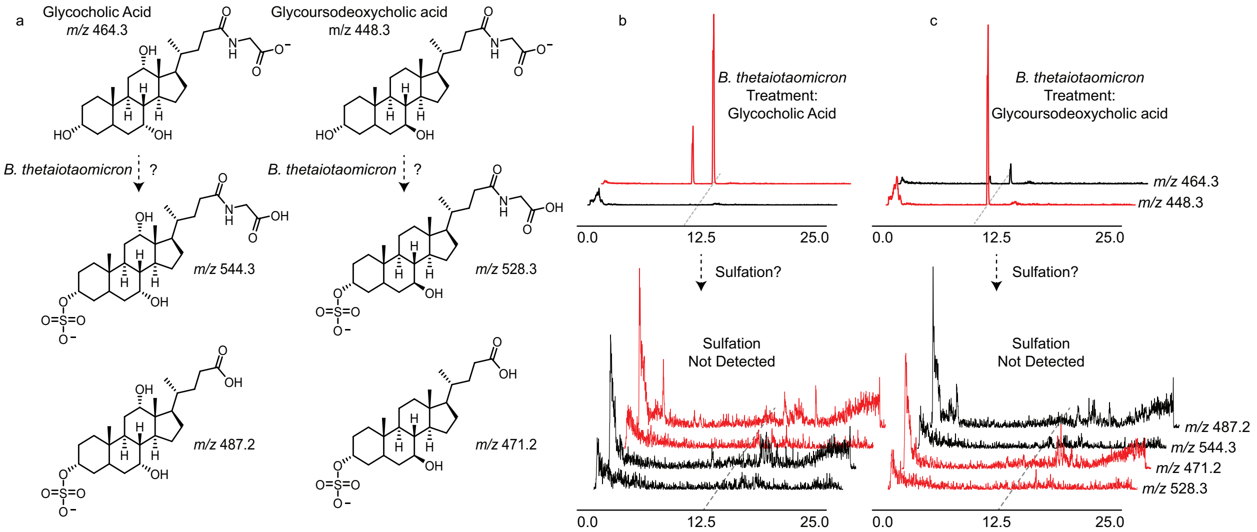

Supplementary Fig. 5 | Conjugated bile acids are not substrates for sulfotransferase in *B. thetaiotaomicron*. a, Schematic of conjugated bile acids tentatively undergoing conversion to their corresponding sulfates via *B. thetaiotaomicron*. Ion chromatograms demonstrating that **b**, glycocholic acid, and **c**, glycoursodeoxycholic acid, are not converted to their corresponding sulfates by *B. thetaiotaomicron*. Red lines indicate the chromatogram and information corresponding to the denoted bile acid.

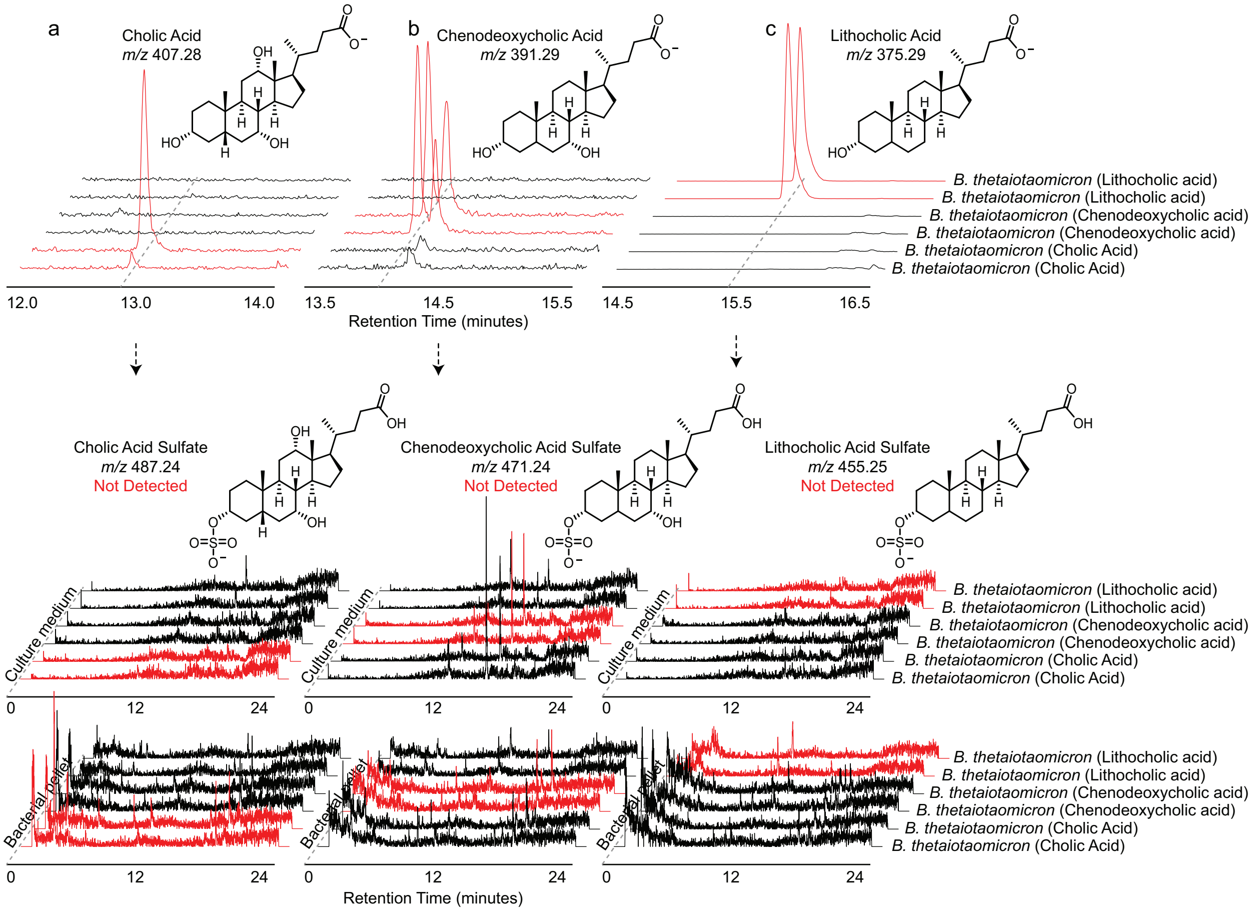

Supplementary Fig. 6 | Primary and secondary bile acids are not substrates for sulfotransferase in *B. thetaiotaomicron*. a, Cholic acid, **b**, chenodeoxycholic acid, and **c**, lithocholic acid are not converted to their corresponding sulfates by *B. thetaiotaomicron*. Red lines indicate the chromatogram and information corresponding to the denoted bile acid.

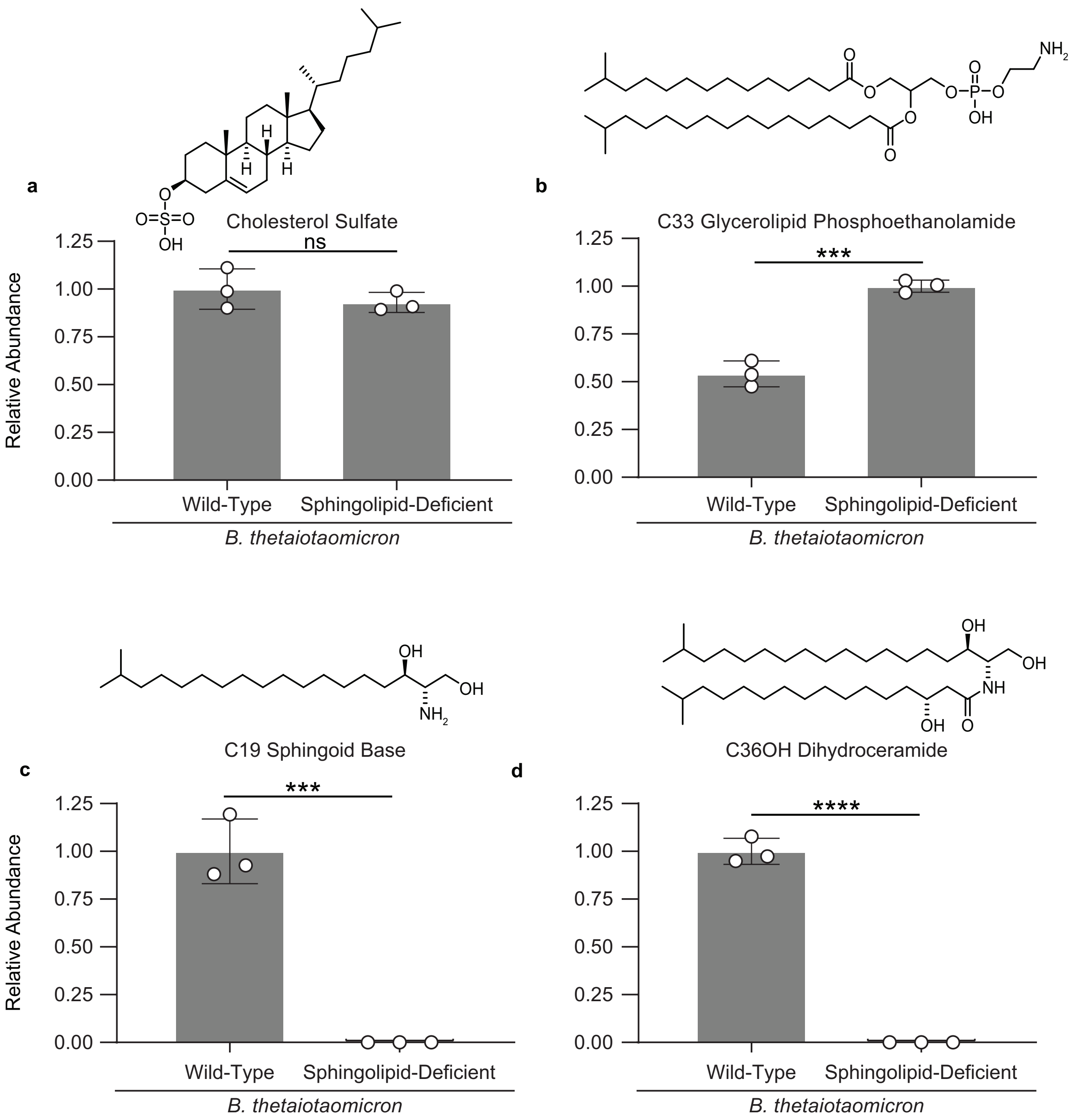

Supplementary Fig. 7 | Cholesterol sulfate production is independent from sphingolipid production in *Bacteroides thetaiotaomicron*. Relative quantification of **a**, cholesterol sulfate, **b**, C33 glycerolipid phosphoethanolamide, **c**, C19 sphingoid base, and **d**, C36OH dihydroceramide from either wild-type or sphingolipid-deficient *B. thetaiotaomicron* cultures. Bar chart values are the mean ± SD. (n = 3 biologically independent cultures per condition).

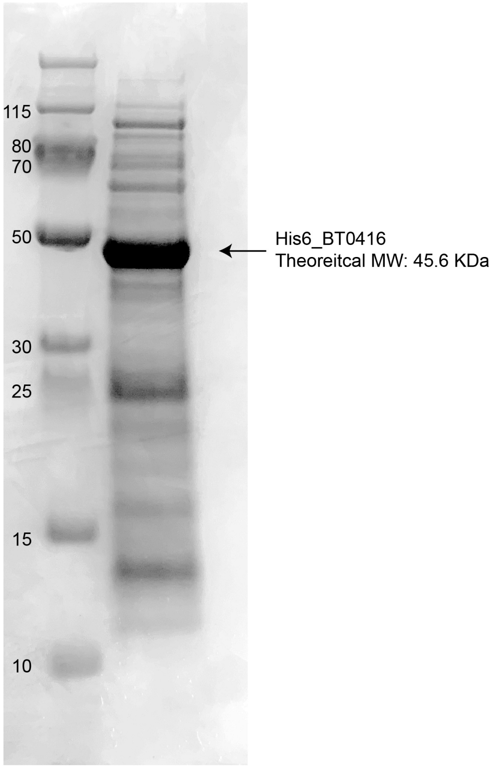

Supplementary Fig. 8 | Coomassie stained SDS-PAGE of partially isolated His6-BT0416 from expression in BL21 *E. coli*.

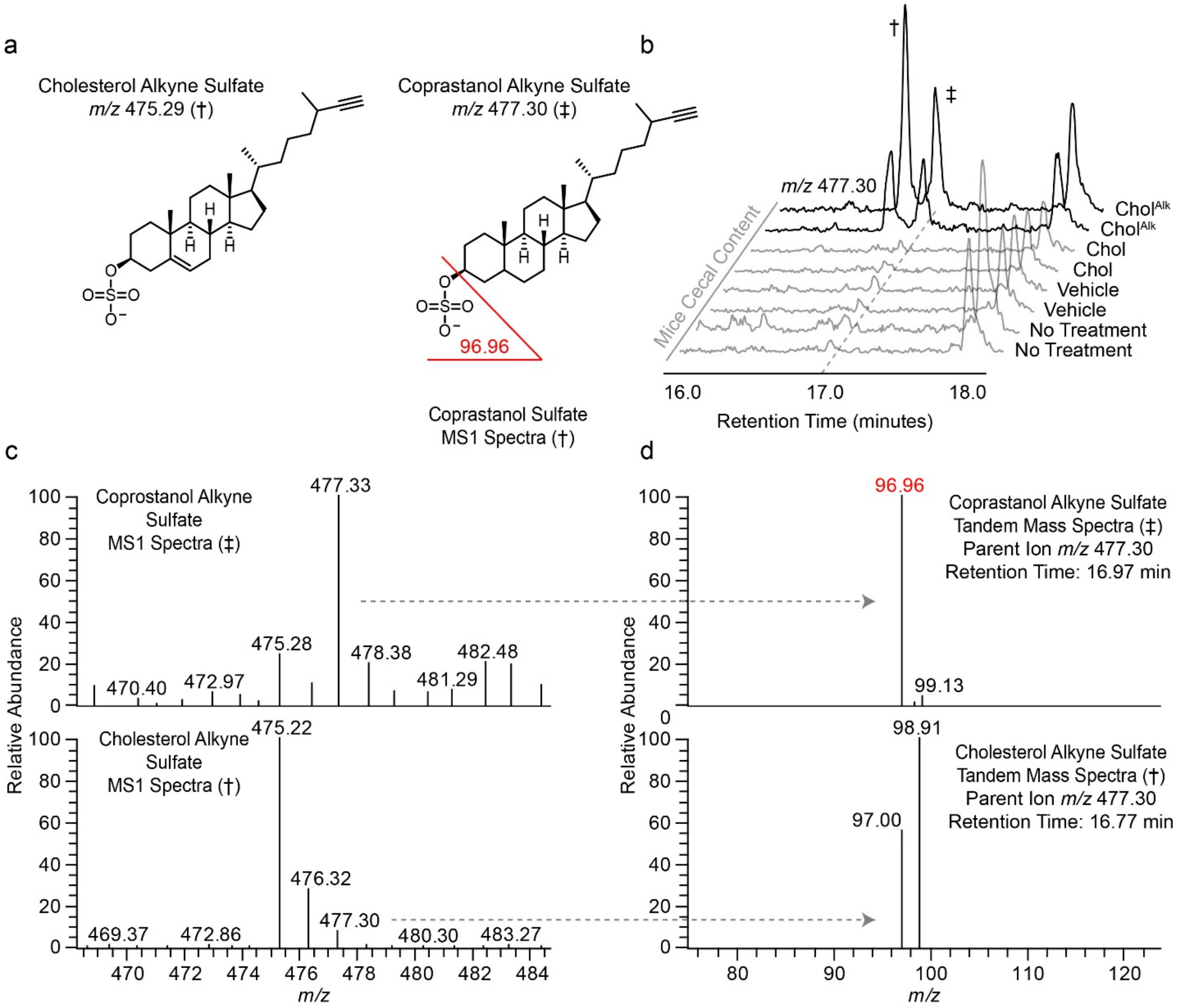

Supplementary Fig. 9 | a, Representative structures, **b**, ion chromatograms, and tandem mass spectra fragmentation patterns of, **c**, coprostanol alkyne sulfate and, **d**, cholesterol alkyne sulfate from cecal content of mice treated with cholesterol alkyne.

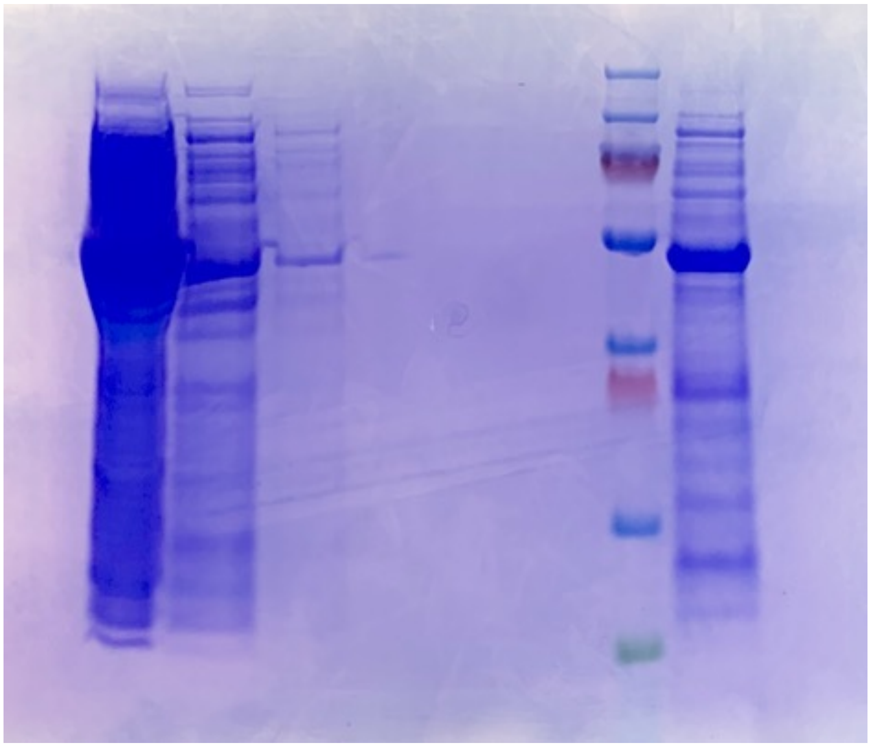

Uncropped blot of Supplementary Figure 8
