# Supplementary material for: Characterization of interactions of dietary cholesterol with the murine and human gut microbiome
Source: Nat Microbiol. 2022 Aug 18;7(9):1390–403. doi: 10.1038/s41564-022-01195-9 (PMC9417993; doi:10.1038/s41564-022-01195-9)
Supplement: Supplementary file 2 — Reporting Summary [file 41564_2022_1195_MOESM2_ESM.pdf]

nature research

Corresponding author(s): Elizabeth Johnson

Last updated by author(s): May 29, 2022

Reporting Summary

Nature Research wishes to improve the reproducibility of the work that we publish. This form provides structure for consistency and transparency in reporting. For further information on Nature Research policies, see our Editorial Policies and the Editorial Policy Checklist.

Statistics

For all statistical analyses, confirm that the following items are present in the figure legend, table legend, main text, or Methods section.

n/a	Confirmed	
☐	☒	The exact sample size (*n*) for each experimental group/condition, given as a discrete number and unit of measurement
☐	☒	A statement on whether measurements were taken from distinct samples or whether the same sample was measured repeatedly
☐	☒	The statistical test(s) used AND whether they are one- or two-sided<br>*Only common tests should be described solely by name; describe more complex techniques in the Methods section.*
☒	☐	A description of all covariates tested
☐	☒	A description of any assumptions or corrections, such as tests of normality and adjustment for multiple comparisons
☐	☒	A full description of the statistical parameters including central tendency (e.g. means) or other basic estimates (e.g. regression coefficient) AND variation (e.g. standard deviation) or associated estimates of uncertainty (e.g. confidence intervals)
☐	☒	For null hypothesis testing, the test statistic (e.g. *F*, *t*, *r*) with confidence intervals, effect sizes, degrees of freedom and *P* value noted<br>*Give P values as exact values whenever suitable.*
☒	☐	For Bayesian analysis, information on the choice of priors and Markov chain Monte Carlo settings
☒	☐	For hierarchical and complex designs, identification of the appropriate level for tests and full reporting of outcomes
☒	☐	Estimates of effect sizes (e.g. Cohen's *d*, Pearson's *r*), indicating how they were calculated

Our web collection on statistics for biologists contains articles on many of the points above.

Software and code

Policy information about availability of computer code

Data collection	Flow cytometry was performed using BD Biosciences Melody Sorter and the real-time data was captured using BD FACSChorus RUO Software v.2.0. LAS X (v 3.6) software from Leica was used for fluorescence microscopy. LC-MS data was acquired using Chromeleon 7.0 Software.
Data analysis	The 16S sequencing data were processed using QIIME2 (v 2020.2) using the DADA2 plugin v2020.2.0 and mapped to SILVA 132 database, and subsequently analyzed in R using the package phyloseq (v1.14.0) according to the calculations stated in the Methods section. The volcano plot for identifying differentially abundant taxa between the two sorted fractions was analyzed and plotted via DESeq2 (v1.36.0) and EnhancedVolcano (1.14.0). For shotgun metagenomic data, Kneaddata (v 0.6.1), Kraken2 (v 2.0.6), KrakenTool, GraPhlAn, and HUMAnN3 (v 3.0) were used to filter and analyze the data, R software was used subsequently for plotting. Images were analyzed using ImageJ FIJI v.1.53h. FlowJo (v 10.6.2) was used for formatting FACS plots. Graphpad Prism 9. Metaboseek 0.9.6 utilizing the XCMS package. Xcalibur v.4.4.16.14 (Thermo Fisher Scientific).

For manuscripts utilizing custom algorithms or software that are central to the research but not yet described in published literature, software must be made available to editors and reviewers. We strongly encourage code deposition in a community repository (e.g. GitHub). See the Nature Research guidelines for submitting code & software for further information.

Data

Policy information about availability of data

All manuscripts must include a data availability statement. This statement should provide the following information, where applicable:

- Accession codes, unique identifiers, or web links for publicly available datasets
- A list of figures that have associated raw data
- A description of any restrictions on data availability

Shotgun metagenomic and V4-16S rRNA sequencing data are deposited as fastq files in the NCBI Sequence Read Archive under Bioproject PRJNA718322. Metabolomics LC-MS data has been deposited at MassIVE with the accession number MSV000087688. Source data is available in the source data files.

Field-specific reporting

Please select the one below that is the best fit for your research. If you are not sure, read the appropriate sections before making your selection.

☒ Life sciences ☐ Behavioural & social sciences ☐ Ecological, evolutionary & environmental sciences

For a reference copy of the document with all sections, see nature.com/documents/nr-reporting-summary-flat.pdf

Life sciences study design

All studies must disclose on these points even when the disclosure is negative.

Sample size	Sample sizes for metabolite tracing experiments in mice were determined from previous work in the lab (Lee et. al. 2020. JLR) which determined that metabolite-interacting microbes could be identified from sorting at least 60,000 microbial cells. Sample size for germ-free/ monocolonization/conventionalization experiments was n=10 per condition and was chosen based off of being able to detect significant differences in hepatic lipid levels in gnotobiotic mice in (Johnson et al. 2020 Nature Communications). Sample size for cholesterol sulfate uptake into hepatic portal vein circulation (n=5 wild type, n=4 null) was based on prior determinations of labeled bacterial lipid uptake into the hepatic portal vein circulation in (Johnson et al. 2020 Nature Communications). All sample sizes are noted in figure legends and methods.
Data exclusions	No data were excluded from the analysis
Replication	Metabolite tracing experiments with cholesterol alkyne for BOSSS workflow (n= 3 mice per condition) were repeated as an independent experiment with confirmation of successful labeling by confocal imaging of cecal contents and congruent results from metagenomic sequencing analysis. For germ-free mouse monocolonization experiments, each bacterial strain was associated with ten germ-free mice and repeated twice successfully with congruent results. Cholesterol sulfate uptake experiments were done once to determine the parameters for measuring uptake (n=2) and then repeated for 5 x wild type and 4 x null gavaged mice.
Randomization	All animals were randomly allocated into groups. Stool from two infants was used for ex vivo culturing to confirm the ability of bacteria in human microbiomes to produce cholesterol sulfate thus allocation into experimental groups was not relevant.
Blinding	Experiments involved the use of modified lipids or strains where knowledge of lipid/strain identity was needed for analysis and sample processing.

Reporting for specific materials, systems and methods

We require information from authors about some types of materials, experimental systems and methods used in many studies. Here, indicate whether each material, system or method listed is relevant to your study. If you are not sure if a list item applies to your research, read the appropriate section before selecting a response.

Materials & experimental systems

n/a	Involved in the study	
☒	☐	Antibodies
☒	☐	Eukaryotic cell lines
☒	☐	Palaeontology and archaeology
☐	☒	Animals and other organisms
☐	☒	Human research participants
☒	☐	Clinical data
☒	☐	Dual use research of concern

Methods

n/a	Involved in the study	
☒	☐	ChIP-seq
☐	☒	Flow cytometry
☒	☐	MRI-based neuroimaging

Animals and other organisms

Policy information about studies involving animals; ARRIVE guidelines recommended for reporting animal research

Laboratory animals	Five- or six-week old excluded flora Female Swiss Webster mice from Taconic Biosciences were used for experiments pertaining to the BOSSS workflow and the evaluation of Bacteroides cholesterol sulfate alkyne in hepatic portal vein blood. Five-week old germfree Female Swiss Webster mice from Taconic Biosciences were gavaged with the appropriate Bacteroides thetaiotaomicron strain and housed in sterile cages until sacrifice to test the effects of Bacteroides cholesterol sulfotransferase activity on host (mouse) cholesterol and cholesterol sulfate levels. Mice were kept in a vivarium where the temperature was kept between 70 - 72°F with a humidity of 47%.
Wild animals	No wild animals were used in this study
Field-collected samples	No samples were collected from the field in this study
Ethics oversight	Cornell University Institutional Animal Care and Use Committee (protocol#2010-0065)

Note that full information on the approval of the study protocol must also be provided in the manuscript.

Human research participants

Policy information about studies involving human research participants

Population characteristics	Healthy infants aged 0 - 12 months
Recruitment	Recruitment was facilitated using approved flyers at health care and child services providers in the greater Ithaca, NY. Infant stool samples were used for ex vivo culturing based on the presence of potential cholesterol-interacting microbes and recruitment bias was not relevant for the completion of these experiments.
Ethics oversight	Cornell Institutional Review Board for Human Subjects Research (protocol# 2007009697)

Note that full information on the approval of the study protocol must also be provided in the manuscript.

Flow Cytometry

Plots

Confirm that:

☒ The axis labels state the marker and fluorochrome used (e.g. CD4-FITC).

☒ The axis scales are clearly visible. Include numbers along axes only for bottom left plot of group (a 'group' is an analysis of identical markers).

☒ All plots are contour plots with outliers or pseudocolor plots.

☒ A numerical value for number of cells or percentage (with statistics) is provided.

Methodology

Sample preparation	Bacteria were isolated from murine cecal content and feces as described in the Methods section by resuspension in PBS and filtration. The extracted bacteria were subsequently covalently bound with fluorophore by click reaction as describe in the Methods section, then were run on the flow cytometer/cell sorter.
Instrument	BD Biosciences Melody Sorter
Software	BD FACSChorus RUO Software v2.0; FlowJo (v 10.6.2) software.
Cell population abundance	Representative population abundances pre- and post-sorting are shown in the SI section of the manuscript. . The purity of samples is indicated in the manuscript, as autofluorescent cells were filtered out of the post-sort population.
Gating strategy	FSC/SSC gates were determined by comparison of cecal/fecal bacterial samples against the control group samples (either no gavage, vehicle or regular cholesterol) to gate in the signal for live bacteria and exclude noise. FSC-H and FSC-A were used to exclude potential doublet cell for minimizing carry-over of bacteria without fluorescence signals. Finally, AF647 gate was developed by using the positive control bacterial strain (Eubacterium coprostaligenes) as described in the Methods main text sections.

☒ Tick this box to confirm that a figure exemplifying the gating strategy is provided in the Supplementary Information.
